# MEK-inhibitor-mediated rescue of skeletal myopathy caused by activating *Hras* mutation in a Costello syndrome mouse model

**DOI:** 10.1242/dmm.049166

**Published:** 2021-11-19

**Authors:** William E. Tidyman, Alice F. Goodwin, Yoshiko Maeda, Ophir D. Klein, Katherine A. Rauen

**Affiliations:** 1 Department of Pediatrics, University of California Davis, Sacramento, CA 95817, USA; 2 UC Davis MIND Institute, Sacramento, CA 95817, USA; 3 Department of Orofacial Sciences and Program in Craniofacial Biology, University of California, San Francisco, CA 94143, USA; 4 Department of Pediatrics and Institute for Human Genetics, University of California, San Francisco, CA 94143, USA

**Keywords:** Costello syndrome, Hypotonia, MEK inhibitor, Myogenesis, RASopathies, Ras/MAPK

## Abstract

Costello syndrome (CS) is a congenital disorder caused by heterozygous activating germline *HRAS* mutations in the canonical Ras/mitogen-activated protein kinase (Ras/MAPK) pathway. CS is one of the RASopathies, a large group of syndromes caused by mutations within various components of the Ras/MAPK pathway. An important part of the phenotype that greatly impacts quality of life is hypotonia. To gain a better understanding of the mechanisms underlying hypotonia in CS, a mouse model with an activating *Hras^G12V^* allele was utilized. We identified a skeletal myopathy that was due, in part, to inhibition of embryonic myogenesis and myofiber formation, resulting in a reduction in myofiber size and number that led to reduced muscle mass and strength. In addition to hyperactivation of the Ras/MAPK and PI3K/AKT pathways, there was a significant reduction in p38 signaling, as well as global transcriptional alterations consistent with the myopathic phenotype. Inhibition of Ras/MAPK pathway signaling using a MEK inhibitor rescued the *Hras^G12V^* myopathy phenotype both *in vitro* and *in vivo*, demonstrating that increased MAPK signaling is the main cause of the muscle phenotype in CS.

## Abstract (precis)

**Summary:** A Costello syndrome (CS) mouse model carrying a heterozygous Hras p.G12V mutation was utilized to investigate Ras pathway dysregulation, revealing that increased MAPK signaling is the main cause of the muscle phenotype in CS.

## INTRODUCTION

The Ras pathway is a well-studied signal transduction pathway important in oncogenesis and is essential for development (Castel et al., 2020). Ras has numerous downstream effector cascades, including the mitogen-activating protein kinase (MAPK) pathway, which is important for cell cycle progression and differentiation, and the phosphoinositide-3-kinase (PI3K; also known as PIK3) pathway, which mediates transcription and anti-apoptotic function through AKT. A group of medical genetics syndromes, known as RASopathies, has germline mutations in components of the Ras/MAPK pathway (Tidyman and Rauen, 2016). RASopathies include neurofibromatosis type 1 (NF1), Noonan syndrome (NS), NS with multiple lentigines, cardio-facio-cutaneous syndrome (CFC), Legius syndrome and Costello syndrome (CS), which collectively represent one of the most common groups of congenital syndromes (Rauen, 2013).

CS is a complex developmental disorder caused by activating heterozygous germline *HRAS* (encodes Harvey rat sarcoma viral oncogene homolog) pathogenic variants (Aoki et al., 2005; Estep et al., 2006). Individuals with CS are born with multiple congenital anomalies, including dysmorphic craniofacial features, cardiac abnormalities, failure to thrive, ectodermal and musculoskeletal anomalies, endocrinopathy, developmental delay and a predisposition to neoplasia, most notably embryonal rhabdomyosarcoma, a tumor of muscle precursor cells (Rauen, 2007). The vast majority of CS individuals harbor a HRAS p.G12S missense mutation, with HRAS p.G12A being the second most common. Rarer pathogenic variants are also seen, including HRAS p.G12V that may be associated with a severe phenotype and is usually incompatible with life (Quezada and Gripp, 2007).

Clinical hypotonia and muscle weakness are universal features in all CS individuals that greatly impact quality of life. CS exhibits the highest degree of skeletal muscle weakness among all the RASopathies (Stevenson et al., 2012). Hypotonia is present at birth, with most individuals having feeding issues. In childhood, motor skills are delayed, muscle weakness and hypotonia persist and a paucity of muscle mass on physical examination is typical. In adulthood, skeletal muscle strength never normalizes to that of the general population, and muscle mass remains decreased throughout life (Gripp et al., 2019). Little is known regarding the myopathy in CS. Muscle biopsies from CS individuals have shown excess muscle spindles (van der Burgt, 2007; de Boode et al., 1996; Selcen et al., 2001), and we reported that skeletal muscle biopsies from CS patients showed excessively small skeletal muscle fibers with a type 2 myofiber predominance (Tidyman et al., 2011).

The role of the Ras/MAPK pathway in skeletal myogenesis has gained importance as a major regulatory component of muscle development, yet its role and how it interacts with other signaling pathways to regulate these processes in a coordinated manner during development is largely unknown. Myogenesis is the process of muscle formation that begins with the commitment of myogenic precursor cells (MPCs) to the myogenic lineage, which is characterized by expression of the transcription factor Pax7 (Buckingham et al., 2006). MPCs subsequently become myoblasts, which continue to proliferate and migrate into the developing musculature (Buckingham et al., 2003). Proliferating myoblasts begin to differentiate by exiting the cell cycle and subsequently fuse to form multinucleated myotubes, or immature muscle fibers/myofibers. During late embryonic and post-natal development, myotubes mature, cluster and hypertrophy, forming mature muscle fibers [for a review, see Bentzinger et al. (2012)]. The embryonic myogenic process and subsequent maturation of muscle is controlled by two classes of muscle-specific transcription factors; the first class consists of the muscle regulatory factors (MRFs) and the second class includes the myocyte-enhancer factor-2 (Mef2) proteins (Black and Olson, 1998; Tapscott, 2005). MRFs, which include MyoD (also known as MYOD1), Myf5, myogenin and MRF4 (also known as MYF6), dimerize with the ubiquitous E proteins (E12 and E47) to activate muscle-specific gene transcription (Maleki et al., 1997). The expression of MyoD and Myf5 in MPCs initiates specification and begins the differentiation process. MyoD is considered to be the key regulator of this process and binds to tens of thousands of genomic locations in muscle, suggesting the potential for broad cellular reprogramming (Cao et al., 2010). During fusion, myogenin and MRF4 are expressed, along with the Mef2 class of transcription factors: Mef2a, Mef2b, Mef2c and Mef2d (Black and Olson, 1998). Both MRF and Mef2 transcription factors act cooperatively to control muscle-specific gene expression during differentiation, during muscle development and in maintaining the skeletal muscle phenotype (Bentzinger et al., 2012; Molkentin and Olson, 1996). Decreased Ras/MAPK pathway activity is also associated with differentiation (Bennett and Tonks, 1997; Heller et al., 2001). However, following myoblast differentiation, Ras/MAPK activity increases and appears to be necessary for continued myotube formation and survival (Li and Johnson, 2006). Ras/MAPK activity is required for the expression of numerous muscle-specific genes during muscle development that are essential for hypertrophic growth (Jo et al., 2009; Scholz et al., 2009; Wu et al., 2000). Although Ras/MAPK pathway activity is necessary, its distinct role in regulation of muscle growth and development remains to be fully elucidated. Of note, several studies have implicated hyperactivation of the Ras/MAPK pathway with the development of skeletal muscle wasting in cancer (Penna et al., 2010; Prado et al., 2012).

A few mouse models have explored the effect of activated Ras/MAPK on skeletal muscle development, including a mouse model for NF1 (Brannan et al., 1994; Kolanczyk et al., 2007), a homozygous knockout model for *Dusp1* (Shi et al., 2010), a mouse model with a homozygous knockout of both *Spry1* and *Spry2* (Michailovici et al., 2014), a cancer mouse model that carries multiple genomic copies of human oncogenic HRAS (Tsuchiya et al., 2005) and, most recently, a mouse model of CFC harboring an intermediate-activating *Braf* allele (Maeda et al., 2021). All of these models demonstrated disrupted muscle development to varying degrees. Mouse models for CS have been developed, but none have examined the skeletal muscle phenotype (Chen et al., 2009; Oba et al., 2018; Schuhmacher et al., 2008). The CS *Hras^G12V^* mouse model has a heterozygous *Hras^G12V^* allele that remains under the control of its endogenous promoter (Chen et al., 2009) and phenocopies the human condition, including low birth weight with reduced survival, craniofacial dysmorphia, cardiac anomalies, hypertension and neoplasia. Based on the marked skeletal muscle weakness in CS individuals, CS provides a powerful and unique model for studying how germline Ras dysregulation in development disrupts skeletal myogenesis. In this study, the *Hras^G12V^* CS mouse model was used to examine skeletal muscle development in the context of Ras/MAPK pathway dysregulation in CS.

## RESULTS

### *Hras^G12V^* mice have decreased skeletal muscle mass and strength

**Fig. 1.**
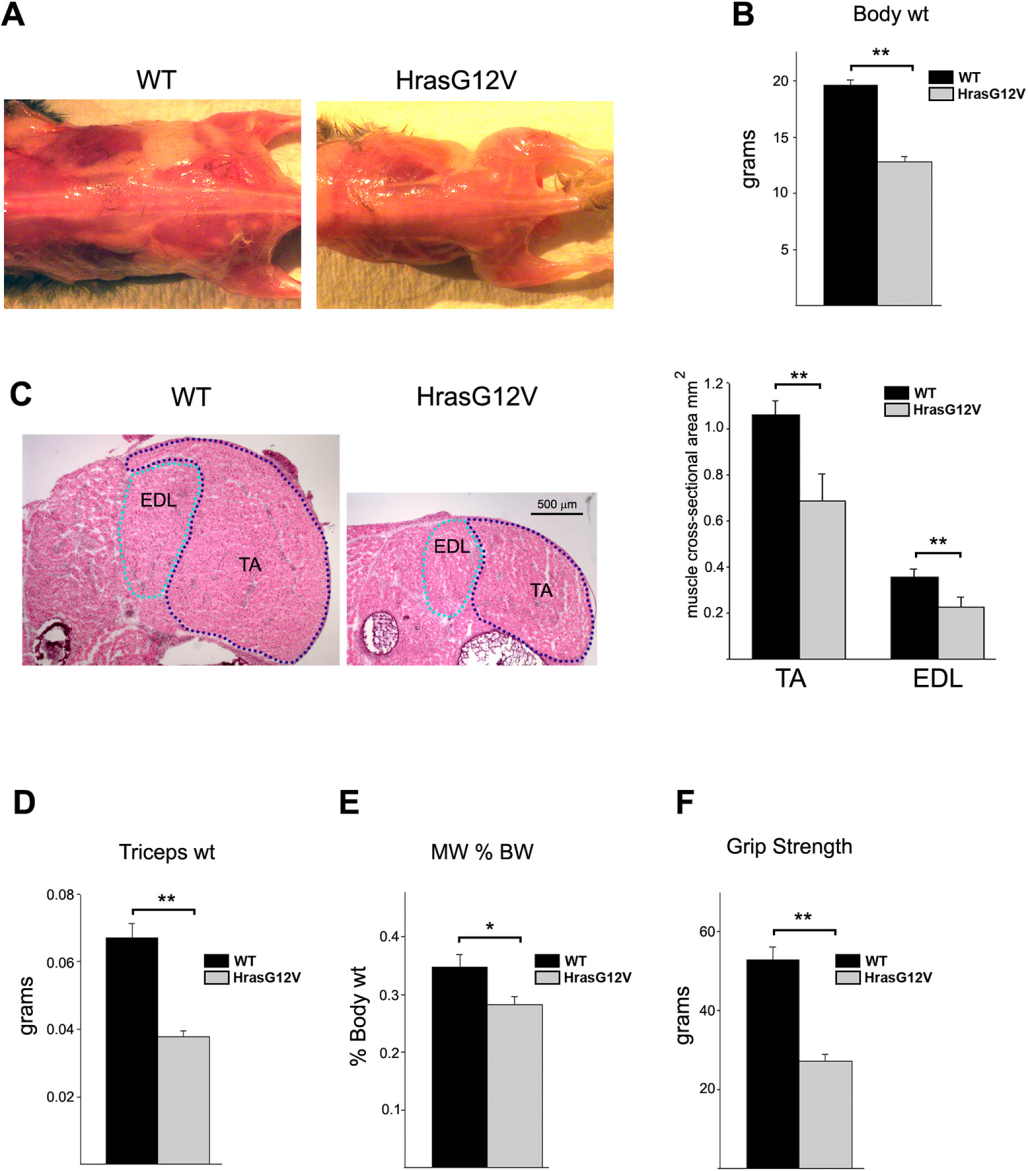
**Examination of the skeletal muscle phenotype of Costello syndrome (CS) *Hras^G12V^* mice compared to that of WT controls.** (A,B) Representative comparison of the gross anatomy of *Hras^G12V^* and wild-type (WT) mice at 1 month of age (A) showed a marked reduction in *Hras^G12V^* musculature and subcutaneous fat, with the average body weight of the mutant mouse significantly less than that of WT (*n*=19; *P*<0.01) (B). (C) H&E-stained cross-section of the lower hindlimb from 5-day-old *Hras^G12V^* and WT mice illustrated the dramatic reduction in muscle girth (5× magnification; scale bar: 500 µm) (left). The overall cross-sectional areas of the *Hras^G12V^* tibialis anterior (TA) and extensor digitorium longus (EDL) muscles were significantly smaller in the *Hras^G12V^* mice compared to WT mice (*n*=5; *P*<0.01) (right). (D,E) The average weight of the *Hras^G12V^* triceps muscle was significantly lower than that of WT triceps muscle (*n*=19; *P*<0.01) (D), with the ratio of triceps weight to body weight (MW % BW) also significantly lower in *Hras^G12V^* mice than in WT mice (*n*=19; *P*<0.05) (E). (F) Functional analysis using grip strength demonstrated significantly weaker forelimb strength in CS *Hras^G12V^* mice compared to WT controls (*n*=15; *P*<0.01). There was no significant difference between male and female WT (*n*=6, *P*=0.81) or male and female CS (*n*=7, *P*=0.56) mice (data not shown). The bars represent the mean±s.e.m. *P<0.05, **P<0.01 (unpaired Student's *t*-test).

To assess the effect of Ras/MAPK pathway dysregulation on skeletal muscle development in CS, a mouse model that contains a heterozygous Hras p.G12V mutation was utilized. As previously described, the CS *Hras^G12V^* mouse model contains a Cre-inducible heterozygous Hras c.35G>T transversion in codon 12 that results in a glycine (G) to valine (V) amino acid substitution (Chen et al., 2009). *Hras^G12V^* mice were noticeably smaller at birth compared to wild-type (WT) littermates and exhibited multiple dysmorphic craniofacial features including a shorter, blunt snout and a rounded cranium (Chen et al., 2009; data not shown). The most prominent phenotypic feature of the *Hras^G12V^* mouse was a dramatic reduction in the overall musculature and subcutaneous fat compared to WT littermates (Fig. 1A). A representative comparison of *Hras^G12V^* and WT at 1 month of age depicted this difference. *Hras^G12V^* mice averaged ∼65% of the weight of the WT mice at 1 month (Fig. 1B). The reduction in muscle size between *Hras^G12V^* and WT was also clearly observed in neonatal mice at 5 days of age (Fig. 1C). Cross-sectional areas of the extensor digitorium longus (EDL) and tibialis anterior (TA) muscles from 5-day-old *Hras^G12V^* mice were ∼65% and ∼67% of the size of the corresponding WT EDL and TA muscles, respectively (Fig. 1C). Additionally, the average weight of the triceps muscle from 1-month-old *Hras^G12V^* mice was significantly lower, 56% of the weight of the WT triceps (Fig. 1D). Comparison of the triceps weight to body weight revealed that the percentage of body weight that the triceps comprised was significantly reduced in the *Hras^G12V^* mouse, at 0.28% compared to ∼0.34% in the WT (Fig. 1E). Thus, the reduction in body weight of the *Hras^G12V^* mice was, in part, due to a reduction in overall muscle mass.

To determine whether a decrease in *Hras^G12V^* muscle mass resulted in a corresponding functional loss in strength, muscle strength was assessed by measuring forelimb grip strength. The grip strength of the *Hras^G12V^* mouse was significantly reduced, averaging 52% of that of WT at 1 month (Fig. 1F). No significant differences were observed between male and female *Hras^G12V^* mice at 1 month with regards to body weight (data not shown), triceps muscle weight (data not shown) and strength.

### Skeletal muscle from *Hras^G12V^* mice exhibits decreased muscle fiber size and reduction in total muscle fiber number

**Fig. 2.**
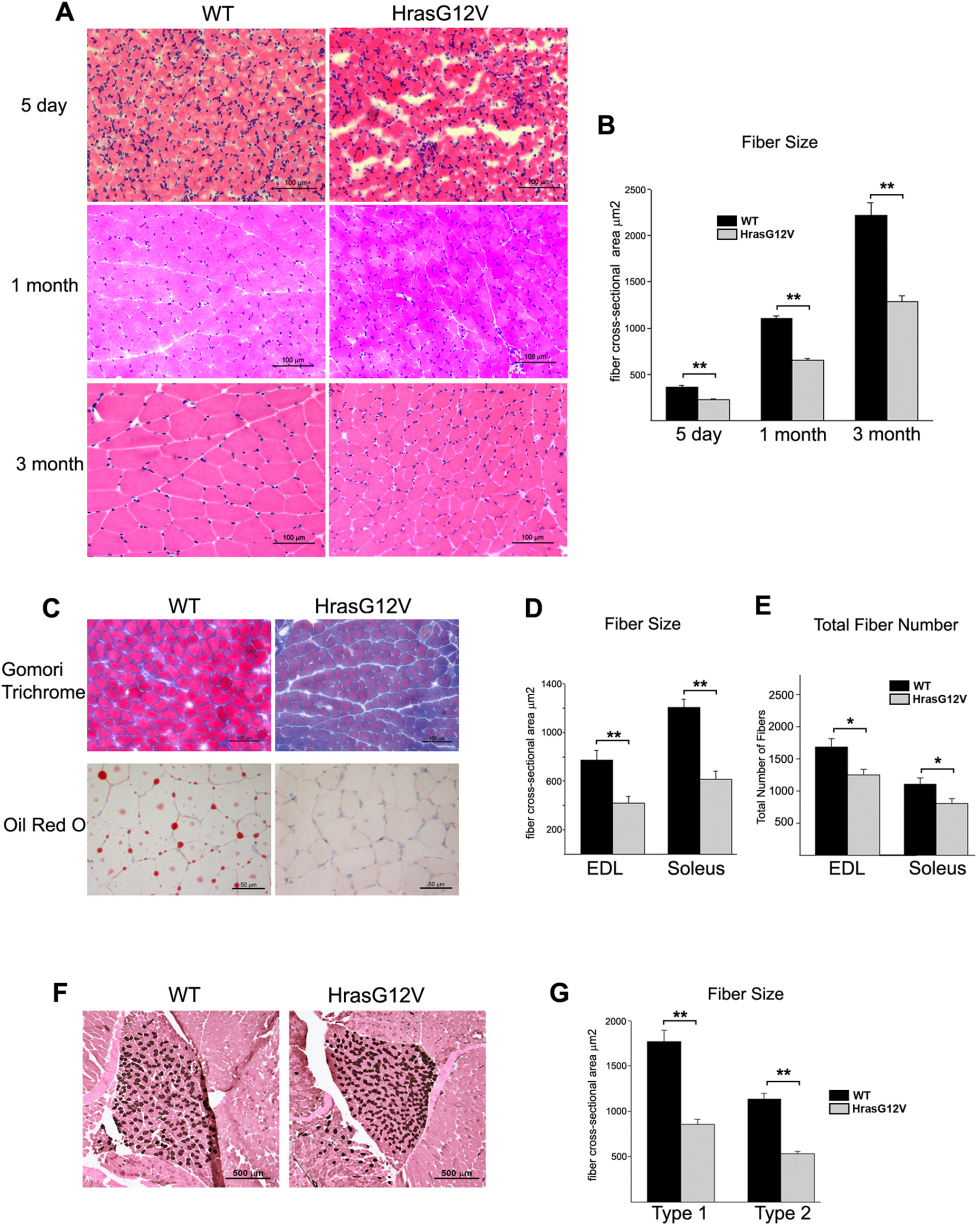
**Histological analysis of gastrocnemius muscle from CS *Hras^G12V^* and WT mice at 1 month of age.** (A) H&E-stained cross-sections of gastrocnemius muscle from 5-day-old, 1-month-old and 3-month-old *Hras^G12V^* mice showed grossly normal morphology (20× magnification; scale bars: 100 µm). (B) Myofiber cross-sectional area was significantly reduced at all three ages examined in *Hras^G12V^* mice compared to age-matched WT controls (*n*=5; *P*<0.01). (C) Gomori trichrome staining revealed excess collagen staining in the *Hras^G12V^* gastrocnemius muscle compared to WT gastrocnemius muscle (20× magnification; scale bars: 100 µm) (top row), whereas Oil Red O staining revealed a complete lack of lipid deposits in the *Hras^G12V^* gastrocnemius muscle compared to WT gastrocnemius muscle at 1 month of age (40× magnification; scale bars: 50 µm) (bottom row). (D) Examination of other muscles including the EDL and the soleus revealed that the myofiber cross-sectional area of each specialized muscle was also significantly smaller in *Hras^G12V^* mice at 1 month of age (*n*=5; *P*<0.01) (D) and had fewer total myofibers (*n*=5; *P*<0.05) (E) compared to that of WT mice. (F,G) ATPase staining at pH 4.3 for type 1 slow fibers in the soleus muscle from WT and *Hras^G12V^* mice (F; 10× magnification; scale bars: 500 µm) suggested that there was no difference between the *Hras^G12V^* and WT type 1 and type 2 fiber counts; moreover, myofiber size analysis showed that both type 1 and type 2 fibers were equivalently reduced in size (G) in the *Hras^G12V^* soleus muscle compared to WT soleus muscle (*n*=5; *P*<0.05). The bars represent the mean±s.e.m. *P<0.05, **P<0.01 (unpaired Student's *t*-test).

In order to determine the primary cause of reduction in *Hras^G12V^* skeletal muscle mass and strength, the gastrocnemius muscle was histologically examined at three ages: 5-day-old neonates, 1-month-old juveniles and 3-month-old adults (Fig. 2A,B). The gastrocnemius muscle has a mixed fiber-type population and, therefore, is representative of musculature in general. Overall, Hematoxylin and Eosin (H&E) staining revealed no apparent signs of histopathology in *Hras^G12V^* muscle. There was no evidence of muscle degeneration, or regeneration, including clusters of small muscle fibers, split fibers or fibers containing centrally located nuclei. There was no indication of vacuoles, inclusions or inflammation. Histological staining of muscle revealed two marked differences between *Hras^G12V^* and WT gastrocnemius muscle at 1 month (Fig. 2C). Gomori trichrome staining showed regions of increased intracellular collagen accumulation in *Hras^G12V^* muscle compared to WT. Oil Red O staining for intramyocellular lipid revealed no evidence of lipid deposits in *Hras^G12V^* muscle, in contrast to WT muscle that showed an abundance of lipid.

The most striking histopathologic difference between *Hras^G12V^* and WT skeletal muscle was that *Hras^G12V^* myofibers were significantly and uniformly smaller in diameter at the three ages examined, measuring ∼60% of the myofiber size of WT controls in cross-sectional area (Fig. 2A,B). The significant reduction in *Hras^G12V^* muscle fiber size was consistent and represented the dominant distinguishing phenotypic feature between *Hras^G12V^* and WT muscle examined. However, the reduction in the muscle mass in the *Hras^G12V^* mouse was due not only to a reduction in the cross-sectional area of myofibers, but also to a reduction in the total number of muscle fibers. The EDL muscle, consisting predominantly of fast-glycolytic myofibers, and the soleus muscle, consisting predominantly of slow-oxidative myofibers (Freitas et al., 2002), were also examined at 1 month of age (Fig. 2D,E). Both muscles showed a significant reduction in overall myofiber size equivalent to that measured in the gastrocnemius. The *Hras^G12V^* EDL and soleus myofiber cross-sectional areas averaged 54% and 52%, respectively, of the size of their WT counterparts (Fig. 2D). Moreover, the total numbers of muscle fibers present at the widest point of muscle girth in *Hras^G12V^* EDL and soleus were significantly decreased, 24% and 22% fewer fibers, respectively, compared to WT controls (Fig. 2E).

Fiber-type analysis by ATPase staining for type 1 (slow), type 2A (fast-oxidative) and type 2B (fast-glycolytic) fibers revealed no significant alterations between *Hras^G12V^* and WT soleus (Fig. 2F) and gastrocnemius muscle (data not shown) at 1 month of age. However, the question of whether the reduction in overall myofiber size impacted both type 1 and type 2 fibers equally was addressed. The cross-sectional area of the type 1 fibers of the soleus muscle in the *Hras^G12V^* mice averaged 54% of the cross-sectional area of the same fibers in the WT (Fig. 2G). Similarly, the type 2 fibers in the *Hras^G12V^* soleus were ∼55% of the size of the type 2 fibers in WT soleus. Therefore, the reduction in fiber size caused by the *Hras^G12V^* mutation was equivalent in both type 1 and type 2 muscle fibers.

### The *Hras^G12V^* mutation inhibits myogenesis during embryonic development

**Fig. 3.**
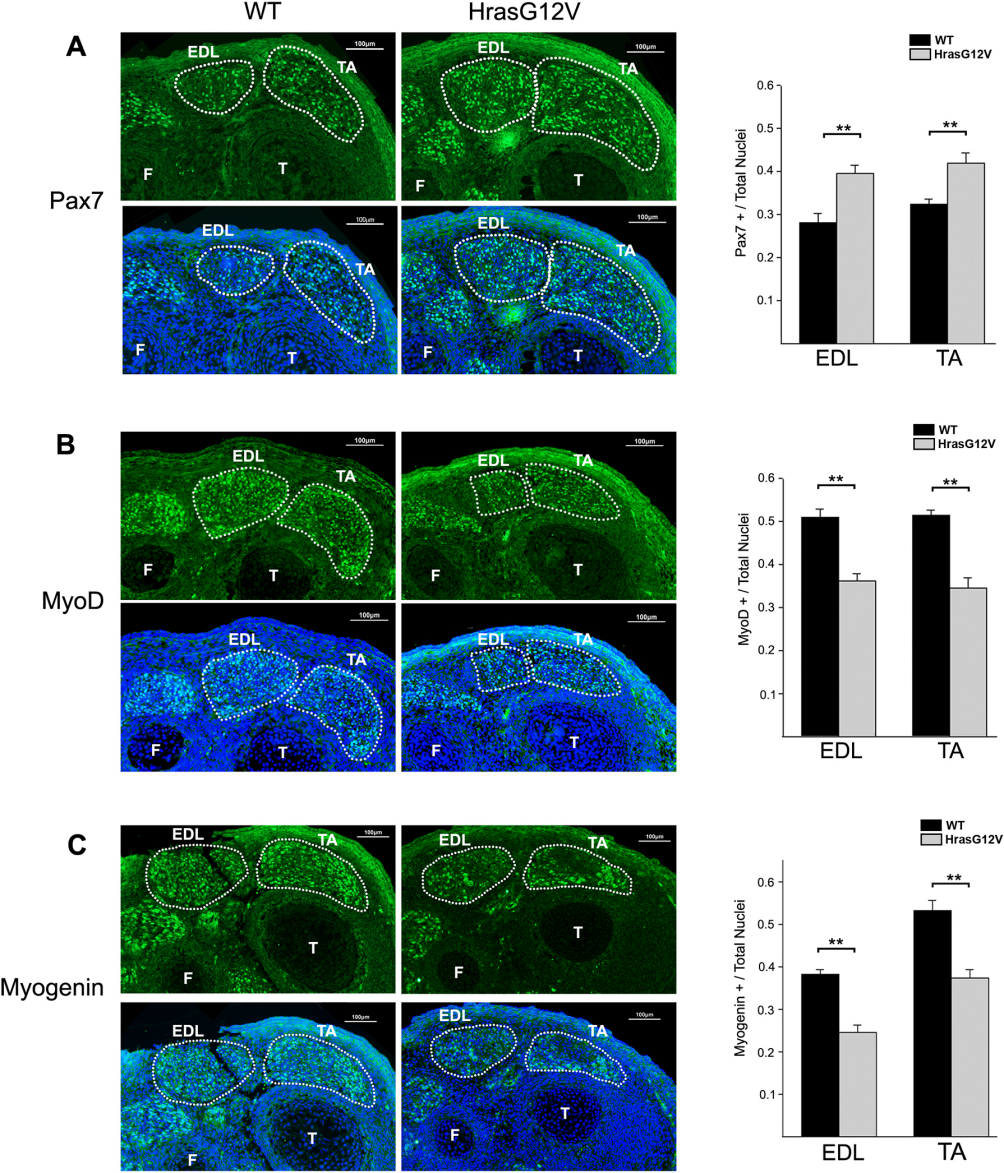
**Embryonic labeling of muscle-related transcription factors.** (A-C) At E14.5, cross-sections of the lower hindlimb from *Hras^G12V^* and WT were immunofluorescently labeled for Pax7 (A), MyoD (B) and myogenin (C) with Alexa Fluor 488 (green). The nuclei were counterstained with DAPI (blue) and merged images are shown. The final images consist of multiple high-resolution fields obtained at 20× magnification (scale bars: 100 µm). The EDL and TA muscles are outlined, with the tibia (T) and fibula (F) labeled for reference. The *Hras^G12V^* embryos had significantly more Pax7-expressing cells in both the EDL and TA muscles at E14.5 compared to WT embryos (*n*=5; *P*<0.01), while the *Hras^G12V^* embryos had significantly fewer MyoD- and myogenin-expressing cells in the EDL and TA muscles compared to WT embryos (*n*=5; *P*<0.01), as shown in the graphs on the right of the images. The bars represent the mean±s.e.m. **P<0.01 (unpaired Student's *t*-test).

In order to identify the mechanisms that account for the effect of the *Hras^G12V^* mutation on embryonic skeletal myogenesis, the expression of several muscle transcription factors was assessed in developing muscles at embryonic day (E)14.5. The expression of Pax7, a marker for proliferation, and the expression of MyoD and myogenin, which are markers of muscle differentiation, were examined in cross-sections of the lower hind leg in EDL and TA muscles at E14.5. The expression of Pax7 was significantly greater in the *Hras^G12V^* muscle compared to WT muscle (Fig. 3A). The *Hras^G12V^* mouse EDL muscle had 29% more Pax7-expressing cells than WT EDL muscle. Likewise, the *Hras^G12V^* TA muscle had ∼23% more Pax7-expressing cells than WT TA muscle (Fig. 3A). In contrast, MyoD, which is initially expressed during myoblast differentiation, was expressed at significantly lower levels in *Hras^G12V^* muscle compared to WT muscle (Fig. 3B). The EDL and TA muscles in the *Hras^G12V^* mouse had 71% and 67% as many MyoD-expressing cells as did the WT EDL and TA muscles. Similarly, myogenin, which is initially expressed during myoblast differentiation and myofiber formation, also had significantly reduced expression in the *Hras^G12V^* muscle compared to WT muscle (Fig. 3C). The *Hras^G12V^* EDL muscle had only 64%, and the TA muscle 70%, of the number of myogenin-expressing cells as the EDL and TA muscles from WT embryos, respectively. Thus, the *Hras^G12V^* muscle contained more Pax7-expressing muscle precursor cells and proliferating myoblasts and far fewer MyoD- and myogenin-expressing cells than did WT muscle, which is indicative of myogenesis inhibition during embryonic development.

### Multiple intracellular signaling pathways are dysregulated in *Hras^G12V^* skeletal muscle

**Fig. 4.**
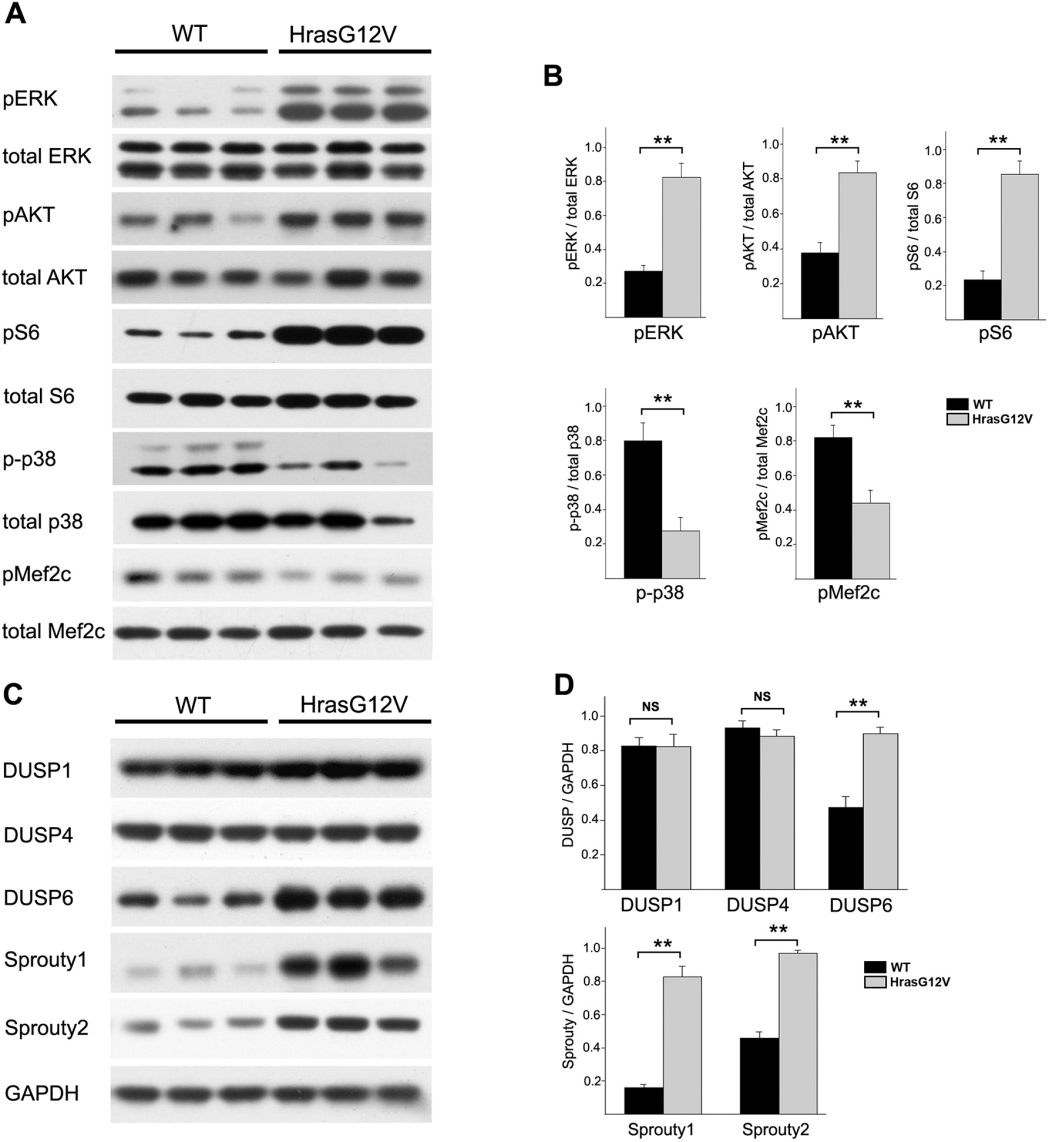
**Western blot analysis of gastrocnemius muscle from 21-day-old *Hras^G12V^* and WT mice.** (A) Western blots comparing the activities of the Ras/MAPK, PI3K/AKT and p38 intracellular signaling pathways between *Hras^G12V^* and WT mice. (B) Both the Ras/MAPK and PI3K/AKT pathways showed significant increase in signaling activity in *Hras^G12V^* gastrocnemius muscle compared to WT gastrocnemius muscle, as demonstrated by increased levels of phosphorylated-ERK (pERK) relative to total ERK and increased level of phosphorylated-AKT (pAKT) relative to total AKT, respectively (*n*=6; *P*<0.01). Likewise, there was a ∼3.6-fold increase in the levels of phosphorylated-S6 (pS6) relative to total S6 in the *Hras^G12V^* muscle compared to WT muscle (*n*=3; *P*<0.01). In contrast, the activity of the p38 MAPK pathway was significantly decreased in *Hras^G12V^* muscle compared to WT muscle, as assessed by a reduced level of phosphorylated (p-p38) compared to total p38 (*n*=6; *P*<0.01). The level of phosphorylated-Mef2c (pMef2c) was also significantly reduced relative to total Mef2c (*n*=6; *P*<0.01). (C,D) The levels of negative regulators of the Ras/MAPK pathway were also assessed by western blotting (C). The levels of Sprouty1, Sprouty2 and DUSP6 showed significant increases in the *Hras^G12V^* muscle compared to WT muscle (*n*=6; *P*<0.01), whereas the levels of DUSP1 and DUSP4 were unchanged (D). The bars represent the mean±s.e.m. **P<0.01; NS, not significant (unpaired Student's *t*-test).

To determine how the Hras p.G12V protein dysregulates skeletal myogenesis, western blot analysis of 21-day-old *Hras^G12V^* and WT gastrocnemius muscle was performed. Because Ras signals through both the MAPK and PI3K/AKT effector pathways, the signaling activities of both pathways were assessed. The *Hras^G12V^* mutation caused a significant increase in Ras/MAPK pathway activity (Fig. 4A). There was a ∼3.2-fold increase in the level of phosphorylated-ERK (pERK) relative to total ERK in *Hras^G12V^* muscle compared to WT muscle (Fig. 4B). This finding supported the hypothesis that hyperactivation of the Ras/MAPK pathway is a primary underlying cause of the skeletal myopathy caused by the *Hras^G12V^* mutation. Additionally, the activity of the PI3K/AKT pathway was assessed by determining the level of phosphorylated-AKT (pAKT) and phosphorylated-p70S6 kinase (pS6). Similar to the effect on the Ras/MAPK pathway, the *Hras^G12V^* mutation resulted in a ∼3-fold increase in the activity of the PI3K/AKT pathway, as measured by pAKT, and a ∼3.6-fold increase in the levels of pS6 relative to total S6 in the *Hras^G12V^* muscle compared to WT muscle (Fig. 4A,B). Thus, the *Hras^G12V^* mutation caused significant activation of both the Ras/MAPK pathway and the PI3K/AKT pathway in skeletal muscle.

In order to confirm the constitutive activation of the Ras/MAPK and PI3K/AKT pathways, the levels of negative regulators of these signaling pathways were examined. Sprouty1 (encoded by *Spry1*) and Sprouty2 (encoded by *Spry2*) are key negative regulators of the Ras/MAPK pathway. Sprouty1 showed a ∼5-fold increase, and Sprouty2 showed a ∼2.1-fold increase, in *Hras^G12V^* muscle compared to WT muscle (Fig. 4C,D). Dual specificity phosphatases (DUSPs) are also negative regulators of the Ras/MAPK pathway as well as the PI3K/AKT pathway. No significant changes were detected in the levels of DUSP1 and DUSP4 in *Hras^G12V^* muscle compared to WT muscle. However, the level of DUSP6 showed a significant ∼2.2-fold increase in *Hras^G12V^* compared to WT (Fig. 4C,D). Increases in Sprouty1/2 and DUSPs are consistent with the constitutive activation of the Ras/MAPK and PI3K/AKT pathway.

The p38 MAPK pathway, which is a key intracellular signaling pathway essential for skeletal muscle growth and homeostasis, is also a target of DUSP6 (Zhang et al., 2011). Phosphorylated-p38 (p-p38) was significantly decreased ∼2.7-fold in *Hras^G12V^* muscle compared to WT muscle (Fig. 4A,B). These data suggest that p38 pathway dysregulation may additionally play an important role in *Hras^G12V^*-mediated skeletal myopathy. Because Mef2 proteins are regulatory targets of p38, the level of phosphorylated-Mef2c (pMef2c) was assessed (Fig. 4A,B). Consistent with a decrease in p38 signaling, the level of pMef2c was significantly decreased ∼2.2-fold in *Hras^G12V^* muscle compared to WT muscle, demonstrating that HRAS activation of skeletal muscle results in dysregulation of multiple signal transduction pathways important to skeletal muscle development and homeostasis.

### The *Hras^G12V^* mutation causes global alterations in skeletal muscle transcription

To examine global alterations in gene transcription in *Hras^G12V^* skeletal muscle, RNA sequencing (RNAseq) analysis was performed using gastrocnemius muscle mRNA from three 21-day-old *Hras^G12V^* and three WT littermates. The rationale for using the gastrocnemius muscle included that (1) it is a large muscle of mixed fiber type, (2) the muscle had significant histological differences in *Hras^G12V^* compared to WT, and (3) this muscle was used in western blot analyses. Direct examination of the sequence reads validated the presence of the heterozygous expression of the *Hras^G12V^* mutation in the mutant mouse. WT *Hras* and *Hras* c.35G>T transcripts were detected in *Hras^G12V^* gastrocnemius muscle at an approximate 1:1 ratio. The *Hras^G12V^* mutation resulted in the differential expression of 1197 genes in mutant muscle compared to WT with a *P*-value of less than 0.05. Of these differentially expressed genes, there were 487 with a fold change of at least 1.5, including 207 transcripts with increased expression (Table S1) and 280 transcripts with decreased expression (Table S2). The global alteration in transcription between the three *Hras^G12V^* and three WT mice was visualized in a volcano plot and heatmap that highlights the consistency of transcriptional alterations between individual samples from the *Hras^G12V^* and WT mice and the overall scope of the transcriptional alterations (Fig. S1).

In order to determine the effect of the *Hras^G12V^* mutation on intracellular signaling pathways, analysis of enriched transcripts using the Kyoto Encyclopedia of Genes and Genomes (KEGG) database was performed on the differentially expressed genes between *Hras^G12V^* and WT (Kanehisa and Goto, 2000). The top four muscle-related KEGG pathways included (1) Ras/MAPK signaling, (2) PI3K/AKT pathway, (3) nuclear factor kappa-light-chain-enhancer of activated B cells (NFκB) and (4) tumor necrosis factor (TNF) (Table 1). The most prominent pattern of differentially expressed genes associated with the Ras/MAPK and PI3K pathways reflected an overall trend that would act to decrease the signaling of these pathways. For example, several negative regulators of Ras signaling, including *Dok3* (encoding a scaffolding protein that sequesters Grb2), *Tbc1d10c* (encoding a RasGTPase activating protein) and *Rasa3* (encoding a RasGTPase activating protein) demonstrated increased relative expression. Likewise, several genes that are positive regulators of Ras/MAPK signaling, or components of the pathway, such as *Egf*, *Fgfl3*, *Fgfr3*, *Rasgfr2* and *Sos2*, showed decreased expression. Genes with relative decreased expression associated with Ras/MAPK signaling also showed overlap with the PI3K pathway. Genes specifically associated with activation of the PI3K pathway, including the focal adhesion/integrin genes *Comp*, *Col11a2*, *Cola1* (also known as *Col1a1*) and *Col24a*, demonstrated relative downregulation. Importantly, KEGG analysis identified numerous relatively increased transcripts in the NFκB and TNF pathways associated with apoptosis and skeletal muscle atrophy in *Hras^G12V^* muscle compared to WT muscle.

**Table 1.**
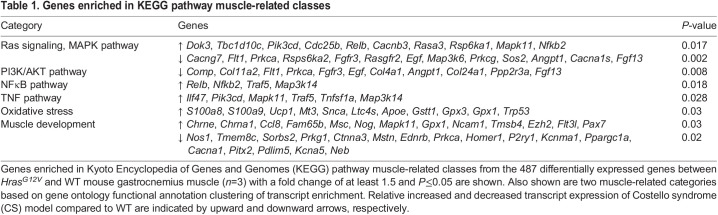
Genes enriched in KEGG pathway muscle-related classes

Gene ontology (GO) functional annotation clustering analysis of the differentially expressed genes was also used to identify categories containing over-represented transcripts in the *Hras^G12V^* muscle compared to WT (Table 1). Two categories of particular relevance to the *Hras^G12V^* muscle phenotype were oxidative stress and skeletal muscle development. Numerous genes indicative of oxidative stress showed increased expression, including *S100a8* and *S100a9*, which showed 8- and 15-fold increase, respectively, in *Hras^G12V^* muscle compared to WT muscle. In addition, metallothionein (*Mt3*) and uncoupling protein 1 (*Ucp1*) were also highly expressed in *Hras^G12V^* muscle. Numerous differentially expressed genes were also associated with skeletal muscle development. Increased transcripts included acetylcholine receptor subunit a1 (*Chrna1*), which showed a 7-fold increase in *Hras^G12V^* muscle, which characteristically occurs during muscle atrophy (Fisher et al., 2017). Musculin (*Msc*), a negative regulator of MyoD transcriptional activity, and *Pax7* were also increased in *Hras^G12V^* muscle compared to WT muscle. In comparison, multiple important genes associated with skeletal muscle development were decreased in *Hras^G12V^* muscle compared to WT muscle, including myoblast fusion factor (*Tmem8c*; also known as *Mymk*) and paired-like homeodomain transcription factor 2 (*Pitx2*), both of which regulate myoblast differentiation (Hernandez-Torres et al., 2017; Quinn et al., 2017). Likewise, myostatin (*Mstn*), a key regulator of skeletal muscle growth and hypertrophy, was decreased in *Hras^G12V^* muscle compared to WT muscle.

In examining transcript alterations using GO functional annotation clustering analysis, of the genes with increased expression, 26 were associated with apoptosis, which is consistent with the finding that numerous genes associated with NFκB and TNF pathway signaling were also increased (Table 2). Several categories were identified with differentially expressed transcripts relating to cellular metabolism. Importantly, numerous mitochondrial genes, including many associated with oxidative phosphorylation, were decreased in *Hras^G12V^* muscle. Moreover, genes associated with carbohydrate and glucose metabolism were also decreased. Genes associated with lipid metabolism were relatively decreased, whereas those specifically related to lipid catabolism were relatively increased. Of particular interest, genes associated with regulation of cellular proliferation were differentially expressed.

**Table 2.**
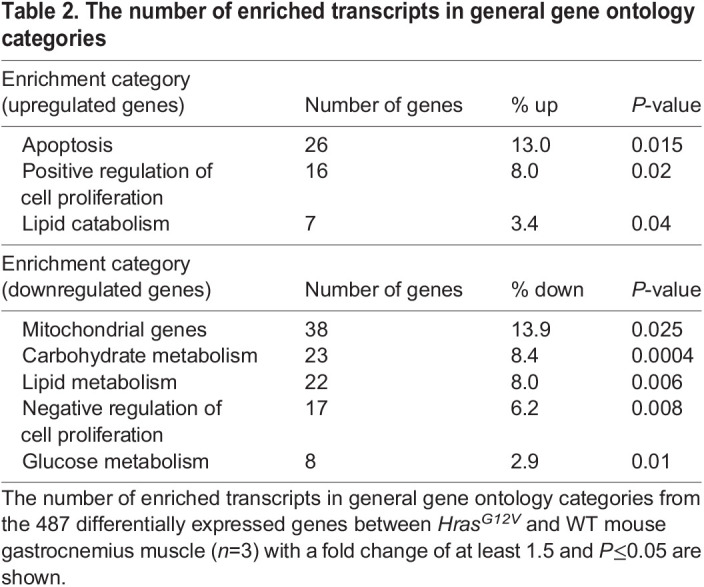
The number of enriched transcripts in general gene ontology categories

### MEK inhibition normalizes both *Hras^G12V^* myoblast differentiation *in vitro* and *Hras^G12V^* skeletal myopathy *in vivo*

**Fig. 5.**
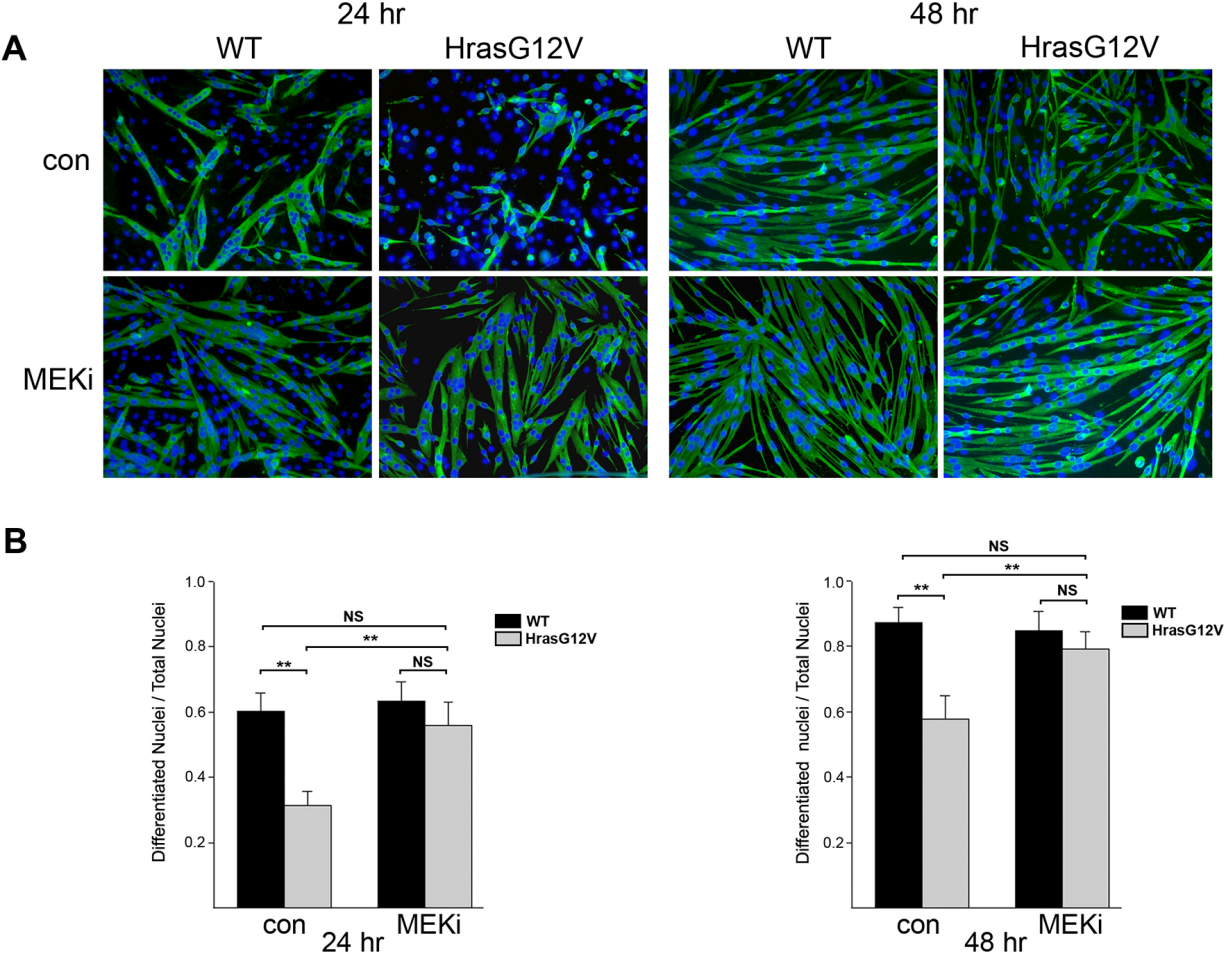
**Treatment of primary myoblast cultures with MEK inhibitor (MEKi).** Primary *Hras^G12V^* and WT myoblast cultures were treated with and without the addition of MEKi PD0325901 to a final concentration of 1 µM. (A) Merged images of immunofluorescently labeled MyHC-expressing cells (green) with DAPI-counterstained nuclei (blue) (10× magnification). (B) At 24 h (left) and 48 h (right) in DM with no MEKi, control *Hras^G12V^* cultures had significantly fewer nuclei in MyHC-expressing differentiated cells, indicating less myotube formation than in WT cultures (*n*=5; *P*<0.01). With the addition of MEKi, the *Hras^G12V^* cultures showed a significant increase in the number of nuclei found in MyHC-expressing differentiated cells and concomitantly showed an increase in myotube formation compared to the untreated *Hras^G12V^* cultures (*n*=5; *P*<0.01). MEKi-treated *Hras^G12V^* cultures exhibited robust elongated myotube formation by 48 h in DM. Importantly, at both 24 h and 48 h in DM, the number of nuclei in MyHC-expressing differentiated cells in the MEKi-treated *Hras^G12V^* cultures was not significantly different from that in either the WT control or WT MEKi cultures (*P*=0.45 at 24 h and *P*=0.16 at 48 h, one-way ANOVA). The MEKi did not significantly alter the differentiation process in the WT myoblast culture. The bars represent the mean±s.e.m. **P<0.01; NS, not significant (unpaired Student's *t*-test).

To determine whether the skeletal myopathy associated with *Hras^G12V^* was intrinsic to muscle development and not a result of neuronal interactions, myogenesis was examined *in vitro* using primary myoblasts derived from neonatal *Hras^G12V^* and WT mice (Fig. S2). Results demonstrated that inhibition of differentiation was inherent to *Hras^G12V^* myoblasts, suggesting that the skeletal myopathy in the CS *Hras^G12V^* mouse model is intrinsic to muscle and represents a ‘true’ myopathy. Thus, in combination with the *Hras^G12V^* mutation resulting in activation of the Ras/MAPK and PI3K/AKT pathways, we sought to determine whether inhibition of these pathways could correct the negative effect of the *Hras^G12V^* mutation on myoblast differentiation. Proliferating myoblasts from *Hras^G12V^* and WT cultures were incubated in differentiation medium (DM), with or without the addition of MEK (also known as MAP2K) inhibitor (MEKi) PD0325901 (Pfizer; 1 µM final concentration). At 24 h in DM, in cultures without MEKi, ∼32% of *Hras^G12V^* nuclei were in differentiated cells expressing myosin heavy chain (MyHC), with very few myotubes present (Fig. 5A,B). With the addition of MEKi to the *Hras^G12V^* myoblast culture, at 24 h, the number of nuclei in differentiated cells increased significantly to ∼58%, along with evidence of myotube formation. Importantly, the number of nuclei in differentiated MyHC-expressing cells in the MEKi-treated *Hras^G12V^* culture was not significantly different from that in the WT myoblast culture at 24 h in DM. The number of nuclei in MyHC-expressing cells in untreated (control) and MEKi-treated WT myoblast cultures averaged 60% and 63%, respectively. Addition of MEKi to WT myoblast culture had no significant effect on myoblast differentiation. By 48 h in DM, control *Hras^G12V^* cultures had, on average, 57% of the nuclei in differentiated cells; however, addition of MEKi increased the number of differentiated nuclei significantly to ∼79%, along with evidence of elongated myotube formation similar to that of WT controls (Fig. 5A,B). Importantly, the number of nuclei in differentiated cells was statistically the same as in the WT myoblast control and MEKi cultures, at 86% and 84%, respectively. Therefore, the addition of MEKi was able to overcome inhibition of *Hras^G12V^* myoblasts differentiation *in vitro*. Because the *Hras^G12V^* mutation also activates the PI3K/AKT pathway, a PI3K inhibitor (PI3Ki; Genentech GDC0941; 1 µM final concentration) was utilized to assess possible correction of *Hras^G12V^* primary myoblast differentiation. The addition of PI3Ki caused myoblast detachment resulting in cell death (data not shown).

**Fig. 6.**
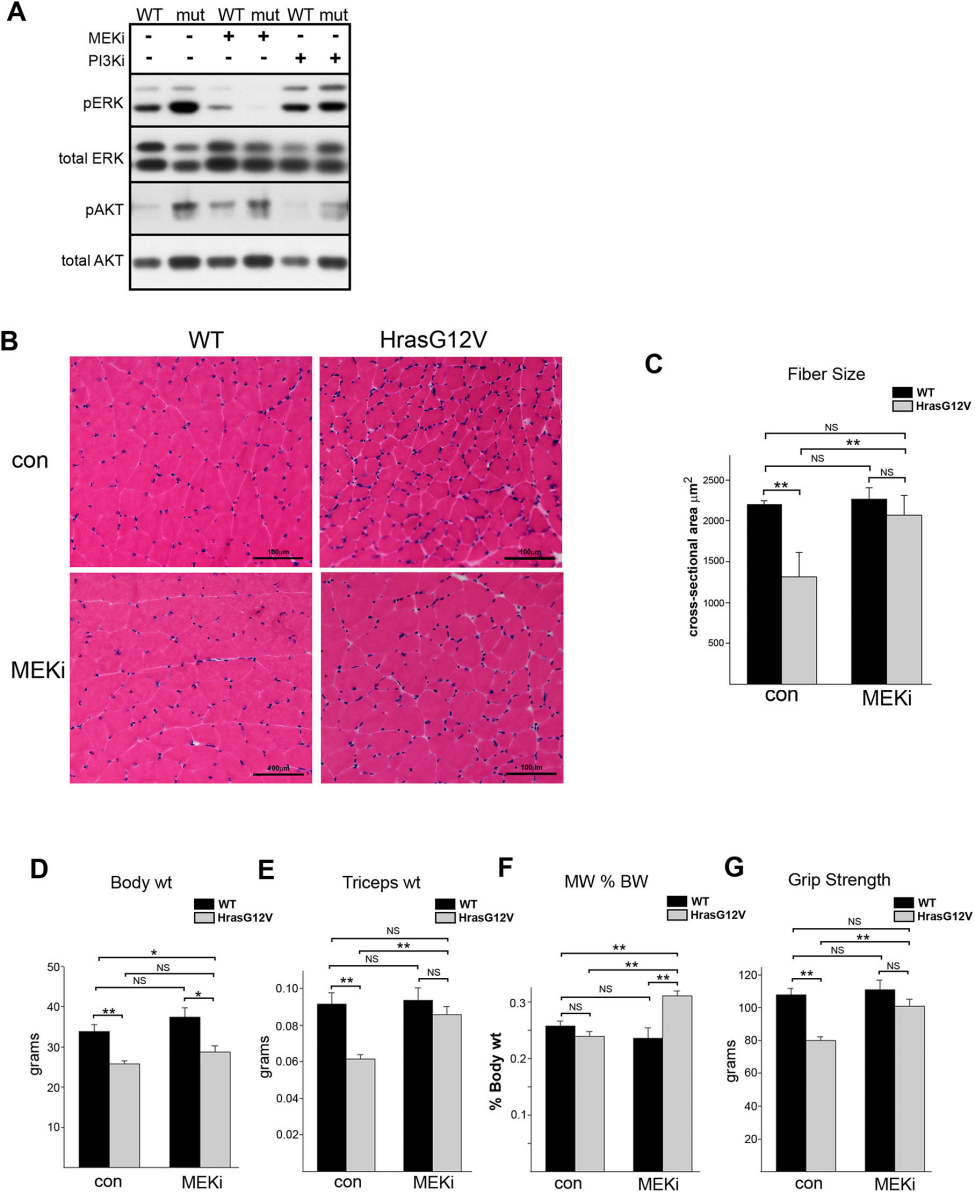
***In vivo* treatment of *Hras^G12V^* mice with MEKi or PI3K inhibitor (PI3Ki).** (A) Representative western blot showing Ras/MAPK and PI3K/AKT pathway signaling in gastrocnemius muscle from *Hras^G12V^* (mut) and WT 3-month-old adult mice treated with either MEKi PD0325901 or PI3Ki GDC0941 for 28 days. The activity of the Ras/MAPK pathway was assessed by the level of pERK and the activity of the PI3K/AKT pathway was assessed by pAKT. Treatment with MEKi specifically reduced pERK levels, and, likewise, treatment with the PI3Ki specifically reduced pAKT levels, in gastrocnemius muscle after 28 days of treatment. (B) H&E-stained gastrocnemius muscle from adult *Hras^G12V^* and WT mice treated with MEKi or vehicle control (con) showed phenotypic rescue of mutant muscle (20× magnification; scale bars: 100 µm). (C) Comparison of gastrocnemius myofiber cross-sectional area between *Hras^G12V^* and WT mice treated with MEKi or vehicle controls showed that treatment of the *Hras^G12V^* mice resulted in a significant increase in gastrocnemius myofiber cross-sectional area compared to that of vehicle control-treated *Hras^G12V^* mice (*n*=5, *P*<0.01, unpaired Student’s *t*-test). Following treatment, there was no significant difference between myofiber size of the WT, WT with MEKi and *Hras^G12V^* with MEKi groups (*n*=5, *P*=0.24, one-way ANOVA). (D-G) Comparison of body weight (D), dissected triceps muscle weight (E), ratio of triceps weight to body weight (F) and forelimb grip strength (G) between MEKi-treated and vehicle-treated (con) WT and *Hras^G12V^* adult mice demonstrated phenotypic rescue in mutant mice after treatment. Treatment with MEKi resulted in significant increases in both triceps muscle weight and grip strength compared to those of vehicle-treated *Hras^G12V^* mice (*n*=5, *P*<0.01, unpaired Student’s *t*-test). The percentage of total body weight of the triceps muscle (MW % BW) also showed a significant increase in the MEKi-treated compared to untreated *Hras^G12V^* mice (*n*=5, *P*<0.01, unpaired Student’s *t*-test). Following treatment, there was no significant difference in either muscle weight (*n*=5, *P*=0.63, one-way ANOVA) or grip strength (*n*=5; *P*=0.30, one-way ANOVA) among WT, WT with MEKi and *Hras^G12V^* mice with MEKi, indicating that 28 days of treatment rescued muscle phenotype and grip strength as measured in this study. WT mice treated with 28 days of MEKi showed no specific difference in body weight, triceps muscle weight, MW % BW or grip strength compared to untreated control WT mice. The bars represent the mean±s.e.m. *P<0.05, **P<0.01; NS, not significant (unpaired Student's *t*-test).

To determine whether inhibition of the Ras/MAPK or PI3K/AKT pathways could rescue the skeletal myopathy caused by the *Hras^G12V^* mutation *in vivo*, either MEKi PD0325901 or PI3Ki GDC0941 was administered daily to 3-month-old adult *Hras^G12V^* and WT mice for 28 days. Western blot analysis of gastrocnemius muscle demonstrated that MEKi or PI3Ki resulted in specific reductions in the Ras/MAPK and PI3K pathway signaling, respectively (Fig. 6A). Impressively, a 28-day treatment with MEKi resulted in phenotypic rescue of gastrocnemius muscle, as indicated by a significant increase in the cross-sectional area of *Hras^G12V^* myofibers compared to vehicle-treated controls (Fig. 6B,C). There was no longer a significant myofiber size difference between *Hras^G12V^* and WT mice. The MEKi-treated *Hras^G12V^* mice showed significant increases in triceps skeletal muscle weight, the percentage of body weight from triceps muscle and grip strength, compared to vehicle-treated *Hras^G12V^* mice (Fig. 6D-G). In addition, following 28 days of MEKi treatment in adult mice, there were no significant differences between the *Hras^G12V^* and WT mice with regards to triceps muscle size and grip strength. The increase in myofiber size, muscle weight and strength following MEKi treatment appeared to be specific to the *Hras^G12V^* mouse, in that no significant phenotypic or functional alteration occurred in the WT MEKi-treated mice compared to WT vehicle-treated controls (Fig. 6B-G). Therefore, treatment with MEKi specifically resulted in phenotypic and functional rescue, as measured in *Hras^G12V^* muscle. In marked contrast to MEKi-treated *Hras^G12V^* mice, *Hras^G12V^* mice treated with the PI3Ki showed no improvement in the *Hras^G12V^* muscle phenotype (Fig. S3). In fact, PI3K treatment had a deleterious effect on both the *Hras^G12V^* as well as the WT muscle phenotypes. PI3Ki administration resulted in significant reductions in muscle fiber diameter, overall muscle size and strength in both the *Hras^G12V^* and WT mice compared to vehicle-treated control mice.

## DISCUSSION

Here, we show that a CS mouse model carrying a heterozygous *Hras^G12V^* activating mutation displays a primary skeletal myopathy that phenocopies features exhibited in CS individuals. The *Hras^G12V^* mutation caused hyperactivation of both the Ras/MAPK and the PI3K/AKT downstream effector pathways of Ras. The mouse skeletal myopathy was characterized by inhibition of embryonic myogenesis and muscle fiber formation at E14.5 and a reduction in overall skeletal muscle mass and muscle strength. The primary cellular phenotypic finding underlying the reduction in muscle mass and strength was a universal reduction in myofiber size. This finding of universal atrophic myofibers was consistent throughout development and persisted in adulthood. In addition to hyperactivation of the Ras/MAPK and PI3K/AKT pathways, there was a significant reduction in the signaling of the p38 MAPK pathway. The *Hras^G12V^* mutation also resulted in global transcriptional alterations in muscle consistent with the skeletal myopathic phenotype. Importantly, inhibition of Ras/MAPK pathway signaling activity using a MEKi was able to rescue the *Hras^G12V^* myopathic phenotype both *in vitro* and *in vivo*.

The CS *Hras^G12V^* mutation caused inhibition of myogenesis during embryonic development. At E14.5, developing hindlimb muscle in the *Hras^G12V^* mouse had significantly more Pax7-expressing cells, and Pax7 expression was still elevated in 21-day *Hras^G12V^* muscle. Pax7 maintains the proliferative state, and its downregulation is necessary for myoblasts to exit the cell cycle (Buckingham and Rigby, 2014). The increase in the number of proliferating cells was associated with a significant decrease in the number of cells expressing MyoD and myogenin in *Hras^G12V^* muscle. MyoD initiates myoblast differentiation and myogenin is required for terminal differentiation to proceed (Conerly et al., 2016). The increase in the number of Pax7-expressing cells and the decrease in the number of MyoD- and myogenin-expressing cells indicates that more myogenic cells remained in the proliferative muscle precursor and myoblast stage, with fewer cells transitioning to a differentiated state. These findings suggest that the inhibition of myogenesis likely represents one of the main disruptive effects the *Hras^G12V^* mutation has on muscle development. This is in accordance with a recent finding that oncogenic Ras inhibits the expression of myogenin in a rhabdomyosarcoma model (Yohe et al., 2018). Myogenic inhibition likely contributes to the primary phenotypic feature of the *Hras^G12V^* mouse skeletal myopathy, that being reduced muscle fiber size and reduced myofiber number, by limiting the population of differentiated myoblasts available for myofiber formation. This inhibition may restrict the final number of mature muscle fibers and may ultimately limit myofiber size due to the principle of nuclear domain, which postulates that myonuclei can only support a finite cytoplasmic volume (Gundersen and Bruusgaard, 2008).

Myogenesis, developmental maturation and homeostasis in mature muscle are regulated by multiple intracellular signaling pathways, including Ras/MAPK, PI3K/AKT and p38 pathways (Knight and Kothary, 2011). The *Hras^G12V^* mutation resulted in constitutive activation of the Ras/MAPK pathway. Previous *in vitro* studies on myogenic cell lines have demonstrated that ERK signaling is necessary for maintaining growth factor-induced myoblast proliferation (Heller et al., 2001). In addition, growth factor withdrawal, which causes downregulation of the Ras/MAPK pathway, initiates the differentiation process (Bennett and Tonks, 1997), while continued growth factor exposure results in continued myoblast proliferation and inhibition of differentiation (Jones et al., 2001; Tortorella et al., 2001). Overexpression of homozygous oncogenic Ras (Olson et al., 1987), and overexpression of constitutively active forms of Raf and MEK1 (Dorman and Johnson, 1999; Weyman and Wolfman, 1998), cause inhibition of myoblast differentiation and myotube formation. Our findings are in accordance with these studies in that an activating heterozygous *HRAS* mutation found in CS patients is capable of inhibiting myoblast differentiation.

The distinct roles of the PI3K/AKT and the p38 MAPK pathways on skeletal muscle growth and development have been extensively studied. The PI3K/AKT pathway is the primary positive regulator of skeletal muscle growth during development and myofiber hypertrophy in mature muscle (Glass, 2010). The *Hras^G12V^* mutation resulted in a ∼3-fold increase in PI3K/AKT signaling. The principal mechanism by which PI3K/AKT activation induces muscle hypertrophy is through activation of the mammalian target of rapamycin (mTOR) (Yoon, 2017). Activation of mTOR leads to phosphorylation and activation of p70S6 kinase (S6), which, in turn, induces protein synthesis and hypertrophy (Glass, 2010). The *Hras^G12V^* muscle exhibited a ∼3.5-fold increase in pS6 levels compared to WT muscle. Despite the fact that mTOR signaling through S6 was significantly increased, the *Hras^G12V^* muscle phenotype consisting of atrophic muscle fibers was the opposite of what would be expected with increased PI3K/AKT activity. In addition to the role of the PI3K/AKT pathway in stimulating muscle hypertrophy, it also directly inhibits the expression of the E3 ubiquitin ligases, MurF1 (encoded by *TRIM63*) and MAFbx/atrogin-1 (encoded by *FBXO32*), which are the primary proteins responsible for muscle atrophy (Bodine and Baehr, 2014; Lee, 2007; Park et al., 2005). It is worth noting that the reduction in myofiber size in the *Hras^G12V^* mouse occurred without increased transcription levels of either Murf1 or MAFbx, as determined by RNAseq.

In contrast to activation of both the Ras/MAPK and PI3K/AKT pathways, the *Hras^G12V^* mutation caused a ∼2.7-fold decrease in the activity of the p38 pathway, as measured by a reduction in the level of p-p38 in *Hras^G12V^* muscle. The p38 MAPK pathway is an essential positive regulator of skeletal muscle development. In contrast to the effect of Ras/MAPK signaling, increased p38 signaling is required for myoblast differentiation to proceed by promoting cell cycle withdrawal (Keren et al., 2006). It is responsible for the downregulation of Pax7 expression required for cell cycle exit (Palacios et al., 2010). Therefore, the activation of the Ras/MAPK pathway and inhibition of the p38 pathway likely act synergistically to inhibit myoblast differentiation and myogenesis in the *Hras^G12V^* mouse.

In addition to its role during myogenesis, activity of the p38 pathway is necessary for muscle-specific gene expression, both during development and in mature muscle (Lluís et al., 2006). In this role, the p38 pathway regulates the transcriptional activity of MyoD by phosphorylating E47 (Lluís et al., 2005). Phosphorylation promotes E47/MyoD hetero-dimer formation, which is necessary for MyoD promoter binding and full transcriptional activity. p38 also regulates the second major class of muscle transcription factors, the Mef2 proteins, by direct phosphorylation, which enhances its transcriptional activity (Wu et al., 2000). The expression of the majority of muscle-specific genes is dependent on both MyoD and Mef2 protein. Thus, the p38 pathway regulates muscle gene expression through control of these two essential transcription factors (Zetser et al., 1999; Zhao et al., 1999). Moreover, Mef2 protein has recently been shown to be one of the primary regulators of muscle fiber size through interaction with the inhibitory myogenic regulatory factor MRF4 (Moretti et al., 2016; Schiaffino et al., 2018). In accordance with the role of the p38 pathway in regulating Mef2, we identified a decrease in pMef2c in *Hras^G12V^* muscle. Therefore, this may represent a critical underlying mechanism by which the *Hras^G12V^* mutation and subsequent decrease in the p38 pathway activity result in the dramatic reduction in myofiber size in the *Hras^G12V^* mouse, which warrants further investigation.

The specific mechanism by which the *Hras^G12V^* mutation caused a reduction in the activity of the p38 pathway remains unclear; however, extensive crosstalk is thought to take place between the Ras/MAPK and p38 pathways (Aksamitiene et al., 2012). Studies using myogenic cell lines have demonstrated that the activity of the p38 MAPK pathway is inversely correlated with that of the Ras/MAPK pathway, which suggests the existence of mutually inhibitory crosstalk (Khurana and Dey, 2002). One mechanism by which p38 activity may be regulated is through the role of the DUSP negative regulators of both the Ras/MAPK and p38 pathways. There was a ∼2.2-fold increase in the level of DUSP6 in *Hras^G12V^* muscle compared to WT muscle. DUSP6 is considered to be a specific negative regulator of the Ras/MAPK pathway, because it preferentially dephosphorylates ERK1/2 (Camps et al., 1998; Caunt and Keyse, 2013). However, DUSP6 also efficiently dephosphorylates p38, but only in the presence of pERK2 (Zhang et al., 2011). DUSP6, pERK2 and p-p38 form a stable complex, which facilitates the dephosphorylation of each substrate (Camps et al., 1998). By this interdependent mechanism, DUSP6 not only controls the activities of ERK2 and p38 but may also mediate mutually inhibitory crosstalk between these two MAPK pathways.

In order to gain insight into global dysregulation of genes in the *Hras^G12V^* muscle, gene transcript alterations in several broad categories were identified using GO functional annotation clustering and gene set enrichment analysis (GSEA). Of the differentially expressed genes that were associated with the *Hras^G12V^* myopathy, two key regulators of muscle development were identified. The first was musculin, an inhibitory basic helix-loop-helix protein that showed a 2.7-fold increase in *Hras^G12V^* muscle (Lu et al., 1999). Musculin competes with MyoD for E-box binding, thereby inhibiting MyoD dependent muscle-specific gene expression (MacQuarrie et al., 2013). In addition, musculin has been shown to inhibit myogenesis and block differentiation in rhabdomyosarcoma cells (Macquarrie et al., 2012; Yang et al., 2009). Of note, embryonal rhabdomyosarcoma is the most common tumor that develops in CS individuals. The second key regulator was myostatin, which showed a 2-fold decrease in *Hras^G12V^* muscle. Myostatin is a member of the TGF-β family that acts as a primary inhibitor of muscle growth and size. Decreased myostatin would tend to induce myofiber hypertrophy (Lee, 2007). Therefore, a decrease in its level may represent a compensatory effect countering the myofiber atrophy in the *Hras^G12V^* mouse.

Global transcriptional alterations indicated dysregulation of energy homeostasis. Thirty-six genes associated with mitochondria were downregulated, consistent with the analysis of muscle biopsies from CS patients that showed deficient mitochondrial enzyme activities (Kleefstra et al., 2011; Tidyman et al., 2011). Among the gene alterations associated with mitochondria, two were notable due to their over-riding effects on mitochondrial function. The first was *Ppargc1a*, which showed a 1.7-fold decrease in expression. It is considered the master regulator of mitochondrial biogenesis, and controls glucose and lipid metabolism (Michael et al., 2001; Puigserver et al., 1998). The second gene was *Ucp1*, which showed an 8.5-fold upregulation in the *Hras^G12V^* muscle. Ucp1 uncouples oxidative phosphorylation, thereby generating heat without ATP production (Chouchani et al., 2019). In addition, both carbohydrate and lipid metabolism were dysregulated in the *Hras^G12V^* muscle. Overall, genes associated with general lipid metabolism showed decreased expression, whereas those specifically associated with lipid catabolism were relatively increased, including *Acat2*, *Apoc1* and *Apoe*. This finding may corroborate the observations of lack of subcutaneous fat and absence of fat deposits in the muscle in the *Hras^G12V^* mouse. In addition, the phenotype of a *Hras^G12S^* mouse model was described as being lean and resistant to obesity despite being fed a high-fat diet (Oba et al., 2018). These findings are consistent with the phenotypic description of CS individuals being very lean with reduced adiposity (Gripp et al., 2019). The dysregulation in energy homeostasis in the *Hras^G12V^* muscle was reflected in the decreased expression of numerous genes associated with carbohydrate metabolism including a 4.2-fold decrease in *Pdk4* expression. PDK4 acts as a key metabolic checkpoint, the downregulation of which facilitates the conversion of pyruvate to acetyl-CoA for oxidative metabolism (Zhang et al., 2014).

Despite the phenotypic consequences of Ras activation in developing skeletal muscle, inhibition of Ras/MAPK signaling was able to rescue the main phenotypic deficits associated with the *Hras^G12V^* skeletal myopathy both in culture and in adult mice. Administration of MEKi normalized myofiber size, muscle weight and strength in *Hras^G12V^* adult mice after a 28-day treatment period. Although we demonstrated that myogenesis was disrupted during embryonic development, we sought to determine whether improvement in the myopathy phenotype could be obtained in adult mice, because most patients are diagnosed with CS in childhood and not prenatally. This is in accordance with perinatal treatment of nursing mice in a NF1 mouse model (Summers et al., 2018). Likewise, treatment with MEKi for Ras-driven oncogenic malignancies alleviated muscle atrophy associated with cachexia in mice and in human patients (Penna et al., 2010; Prado et al., 2012). These data, combined with the ability of the MEKi to rescue *Hras^G12V^* myoblast differentiation *in vitro*, sets forth a future hypothesis that inhibition of embryonic myogenesis may be amenable to correction, given the correct therapeutic developmental window (Anastasaki et al., 2012).

The CS mouse model, which harbors a *Hras^G12V^* mutation, displayed a skeletal myopathy that robustly phenocopies the myopathic features seen in CS individuals. In this study, the myopathy was characterized by inhibition of myogenesis and decreased muscle mass and strength, which resulted from a reduction in myofiber size and total myofiber number. The *Hras^G12V^* mutation resulted not only in activation of both the Ras/MAPK and PI3K/AKT pathways and inhibition of the p38 pathway but also caused a global dysregulation of transcriptional activity. We recently described a similar, albeit less severe, skeletal myopathy in a CFC mouse model due to an intermediate-level *Braf^L597V^* activating mutation (Maeda et al., 2021). Both *Hras^G12V^* and *Braf^L597V^* mutations activated the Ras/MAPK pathway and inhibited the p38 pathway. The Braf mutation, directly downstream of Ras, did not activate the PI3K/AKT pathway. These data suggest that the primary driving alteration for the development of the skeletal myopathy in these two RASopathy models is hyperactivation of the Ras/MAPK pathway. In support of this conclusion is the finding that inhibition of the Ras/MAPK pathway using a MEKi corrected many aspects of the skeletal myopathy in the *Hras^G12V^* mouse, whereas use of a PI3Ki produced additional muscle atrophy and loss of strength. Given the multitude of studies that have shown that the PI3K/AKT pathway is essential for skeletal muscle growth and homeostasis, it is not surprising that inhibition of this pathway would have a detrimental effect (Knight and Kothary, 2011). It is clear in this study that these intracellular signaling pathways act in concert with multiple mechanisms of interaction to orchestrate muscle development and regulation of the adult muscle phenotype. Future studies will be needed to decipher the role and interactions of these pathways during normal muscle development and in disease states. This study also helps guide the development of therapeutic approaches to treat RASopathies, remembering that in the treatment of developmental disorders, one would like to ‘dial down’ MAPK activity as opposed to fully abrogating pathway activity (Rauen et al., 2011). Finally, this study may be applicable to future studies regarding the function of the Ras/MAPK pathway in other skeletal muscle disorders, including muscle-related cancers such as rhabdomyosarcoma.

## MATERIALS AND METHODS

### CS mouse model breeding and genotyping

All animal procedures were performed according to institutional guidelines approved by the Animal Care and Use Committees at University of California Davis and the University of California San Francisco. The CS *Hras^flG12V/flG12V^* (Hras^tm1Jaf^, MGI ID 3845022) and Caggs-Cre [Tg(CAG-cre)1Kymm, MGI ID 4418695] breeding mice were generous gifts from Dr James Fagin at Memorial Sloan-Kettering Cancer Center, New York, NY, USA. The CS *Hras^flG12V/flG12V^* mouse was generated using a flox-and-replace strategy to produce a Cre recombinase-dependent exchange of expression between the WT *Hras* allele and the inserted *Hras^G12V^* allele as previously described (Chen et al., 2009). Expression of Cre results in the excision of the WT allele and expression of the *Hras^G12V^* allele under the control of its intrinsic promoter. The *Hras^flG12V/flG12V^* and Caggs-Cre mice were both bred on a background strain of 50% SWR/J, 25% 129S1/SvImJ and 25% C57BL/6J. All animal experiments were carried out using the first offspring from breeding a *Hras^flG12V/flG12V^* female mouse and a Caggs-Cre+/− male mouse. The resulting genotypes of the offspring used in this study were Caggs-Cre+/*Hras^G12V/+^* (*Hras^G12V^*) and Caggs-Cre−/*Hras ^flG12V^*^/+^ (WT). Genotyping of the *Hras^G12V^* and WT mice was carried out as previously described (Chen et al., 2009).

### Mouse grip strength measurement

Mouse grip strength testing was done using a digital force strain gauge with a 200-g range (±0.01 g; FGV-XY Electromatic Equipment Company). The force gauge was attached to a rigid wire grid consisting of 1-cm squares for ease of grasp to evaluate forelimb strength *in vivo*. Mice were suspended above the grid by the base of their tail until they firmly grasped the grid with their fore limbs, and then the mice were gently pulled horizontally until they released their grasp. The maximum force required for grip release was recorded. Three separate measurements were made per mouse with a 1-min rest period between tests.

### Histology, microscopy and embryonic immunofluorescent labeling

Skeletal muscle was snap-frozen by immersion in isopentane chilled in liquid nitrogen. Frozen muscle was embedded in optimum cutting temperature embedding medium (Tissue-Tek^®^ OCT Compound, Sakura, #4583) and sectioned to 8 µm using a cryostat. Muscle sections were stained with H&E, Gomori trichrome, ATPase (pH 4.3 and 9.4) and Oil Red O using standard protocols (Dubowitz and Sewry, 2007). Stained sections were imaged using a Nikon Eclipse CI-L upright microscope and NIS Elements Viewer software (Nikon Instruments).

Immunofluorescent labeling of embryonic muscle was performed as previously described (Maeda et al., 2021). In brief, the hind limbs from E14.5 embryos were fixed in paraformaldehyde, cryoprotected in sucrose buffer overnight and frozen in OCT embedding medium. Tissue was cross-sectioned using a cryostat to 8 µm. Antigen retrieval was performed by incubation in citric acid buffer and sections were blocked using MOM^TM^ blocking reagent (Vector Labs, #MKB-2213) and 5% goat serum in phosphate buffered saline (PBS). Sections were incubated with primary antibody against Pax7 [Developmental Studies Hybridoma Bank, University of Iowa (DSHB), 1:50], MyoD (BD Pharmingen, #554130, 1:50) or myogenin (DSHB, #F50, 1:50) overnight and subsequently labeled with secondary antibody goat anti-mouse IgG1 conjugated to Alexa Fluor 488 (Invitrogen, #A21121, 1:1000) at room temperature (RT). The nuclei were counterstained with 4′,6-diamidino-2-phenylindole (DAPI). Muscle fiber cross-sectional area measurements were made on H&E-stained sections using ImageJ software (Schneider et al., 2012). At least three random fields were imaged, each myofiber fiber was outlined, and the cross-sectional area was measured using the ImageJ area measurement tool. On average, 100 fibers were measured per field. For fiber number measurements, the total number of muscle fibers, at the point of widest girth, were counted in the EDL and soleus muscles. Muscles were serially sectioned at the point of widest girth to a thickness of 8 µm. For each muscle, the total number of muscle fibers were counted using ImageJ in three sections, ∼200 µm apart.

### Primary myoblast culturing and immunofluorescent labeling

Primary myoblast cultures were established from the limb muscles of 3- to 5-day-old neonatal mice (Rando and Blau, 1994). Primary myoblasts were grown on collagen-coated plates in growth medium (GM) consisting of Ham's F-10 (Gibco) supplemented with 20% non-heat inactivated fetal bovine serum (Invitrogen), penicillin/streptomycin (100 U/ml) and 2.5 ng/ml fibroblast growth factor (Roche, #111231490001). All experiments were carried out on cultures at a passage number of ∼8-9. DNA sequencing was carried out on myoblasts used in experiments to verify *Hras* status. Myoblasts were induced to differentiate by changing the medium to DM, consisting of Dulbecco's modified Eagle medium (Gibco) containing 5% horse serum and penicillin/streptomycin (100 U/ml). For immunofluorescent labeling of primary myoblast cultures, dishes were rinsed with PBS and fixed in 100% methanol for 30 min at −20° C and 70% ethanol for 5 min at RT. Dishes were labeled with anti-MyHC primary antibody MF20 (DSHB, 1:1000) and secondary antibody goat anti-mouse IgG conjugated to Alexa Fluor 488 (Invitrogen, #A11011, 1:1000).

### Western blot analysis

Gastrocnemius muscle samples were dissected and snap frozen in liquid nitrogen. Approximately 2-5 mg of skeletal muscle was homogenized in lysis buffer consisting of 20 mM Tris-HCl (pH 7.5), 150 mM NaCl, 1 mM Na_2_EDTA, 1 mM EGTA, 1% Triton X-100, 2.5 mM sodium pyrophosphate, 1 mM beta-glycerophosphate, 1 mM Na_3_VO_4_, 10 μg/ml leupeptin and phosphatase inhibitor cocktail PhosSTOP (Roche, #11836153001). The lysates were cleared by centrifugation and soluble protein quantified using a Bradford assay. Polyacrylamide gel electrophoresis was carried out on 5 µg protein using a NuPAGE Novex 4-12% Bis-Tris gradient gel (Invitrogen) and transferred to PVDF membrane. Western blot antibody hybridization was carried out using standard protocols and visualized using enhanced chemiluminescent detection reagent (Pierce, #32109). Primary antibodies used to assess intracellular signaling levels were from Cell Signaling Technology (Danvers, MA, USA) and included anti-phosphorylated-p44/42 (p-ERK1/2, #9101, 1:1000), anti-p44/42 (total ERK1/2; #9102, 1:1000), anti-pAKT (Ser473; #4060S, 1:500), anti-total AKT (#9272, 1:1000), anti-p38 (#4511, 1:500), anti-total p38 (#9212, 1:1000), anti-pS6 (#4858, 1:1000), anti-total S6 (#2217, 1:1000), anti-Sprouty1 (#13013, 1:2500) and anti-Sprouty2 (#14954, 1:1000). Additional antibodies used included anti-DUSP1 (Santa Cruz Biotechnology, #sc-1199, 1:500), anti-DUSP4 (Santa Cruz Biotechnology, #sc-10797, 1:500), anti-DUSP6 (Abcam, #ab76310, 1:1000), anti-pMef2c (Abcam, #ab78888, 1:1000), anti-total Mef2c (Abcam, #ab64644, 1:1000) and anti-GAPDH (Ambion, #AM4300, 1:100,000). Western blot hybridizations were quantified using ImageJ Gel Analyzer Tool (Schneider et al., 2012).

### RNAseq and analysis

Gastrocnemius muscle from 21-day-old *Hras^G12V^* and their respective WT littermates was dissected and immediately frozen in liquid nitrogen. mRNA was extracted using an RNeasy Universal Kit (Qiagen) and quality was assessed using a Bioanalyzer RNA 6000 Nano (Agilent Technologies). Libraries for RNAseq were prepared using a KAPA Stranded mRNA-Seq Kit (Roche). RNAseq was carried out using an Illumina HiSeq3000 sequencer (Illumina, San Diego, CA, USA) to ∼50×10^6^ single ends, 50 bp reads per sample. Analysis of the RNAseq data was performed using a TopHat-Cufflinks pipeline (Trapnell et al., 2012). In brief, sequence read alignment/mapping was done with TopHat. Expression analysis consisting of transcript assembly, estimate of transcript abundance/expression, normalization and differential expression analysis was performed using Cufflinks. Normalized fragments per kilobase of transcript per million mapped read (FPKM) values (Cuffnorm output) were imported into GeneSpring GX (Agilent Technologies) for the initial filtering of the data (e.g. removal of entities having negligible or no expression in all samples), to perform statistical testing of differential expression (e.g. mutant versus WT, unpaired Student's *t*-test, false discovery rate-corrected *P*-value<0.05, ±1.5-fold change cut-off), hierarchical clustering and results visualization of volcano plot and heat map. Gene enrichment analysis on the resulting differentially expressed gene lists was carried out using Database for Annotation, Visualization and Integrated Discovery (DAVID; v6.8, https://david.ncifcrf.gov/tools.jsp) with *P*-values calculated using Fisher's exact test to measure the gene enrichment in annotation terms. In addition, GSEA was also used as a comparison tool to analyze gene sets that have been significantly enriched or depleted in the mutant as compared to the WT (Subramanian et al., 2005). GSEA was performed using the filtered, normalized FPKM data and gene sets from the Molecular Signatures Database (Liberzon et al., 2015).

### *In vitro* and *in vivo* inhibitor experiments

Two signal transduction pathway inhibitors were used in this study. PD0325901 (Pfizer; a kind gift from Martin McMahon, Huntsman Cancer Institute, University of Utah, Salt Lake City, UT, USA) is a selective and non ATP-competitive MEKi, and GDC-0941 (a kind gift from Genentech) is a highly selective class I PI3Ki specific for p110α and p110β subunits (Folkes et al., 2008). For *in vitro* primary myoblast culture inhibitor experiments, 1 µM final concentration of MEKi PD0325901 or PI3Ki GDC-0941 was added to low-serum DM until cells were harvested. For *in vivo* inhibitor treatment, 3-month-old *Hras^G12V^* and 3-month-old WT littermates were administered either MEK inhibitor at 12.5 mg/kg (Trejo et al., 2012) or PI3K inhibitor at 75 mg/kg once a day (Folkes et al., 2008) for 28 days by oral gavage. Control mice were given vehicle only. Each study arm had five mice per group, except for the PI3Ki arm, which had three mice. The inhibitor compounds were formulated in 0.5% (w/v) hydroxypropyl methyl cellulose, which also served as the vehicle (Sigma-Aldrich).

### Statistical analysis

Statistical analysis comparing two means was performed using unpaired Student's *t*-test. Statistical analysis of multiple means was performed by one-way analysis of variance (ANOVA). The *P*-values were calculated and statistical significance evaluated, as indicated as *P*-values less than 0.05. The graph bars represent the sample mean±s.e.m. The sample size number (*n*) is indicated for each experiment in the figure legends. GO and KEGG analysis *P*-values were calculated using Fisher's exact test.

## Supplementary Material

Supplementary information
