# Supplementary material for: MEK-inhibitor-mediated rescue of skeletal myopathy caused by activating Hras mutation in a Costello syndrome mouse model
Source: Dis Model Mech. 2021 Nov 19;15(2):dmm049166. doi: 10.1242/dmm.049166 (PMC8617311; doi:10.1242/dmm.049166)
Supplement: Supplementary information [file dmm-15-049166-s1.pdf]

Supplemental Figure 1

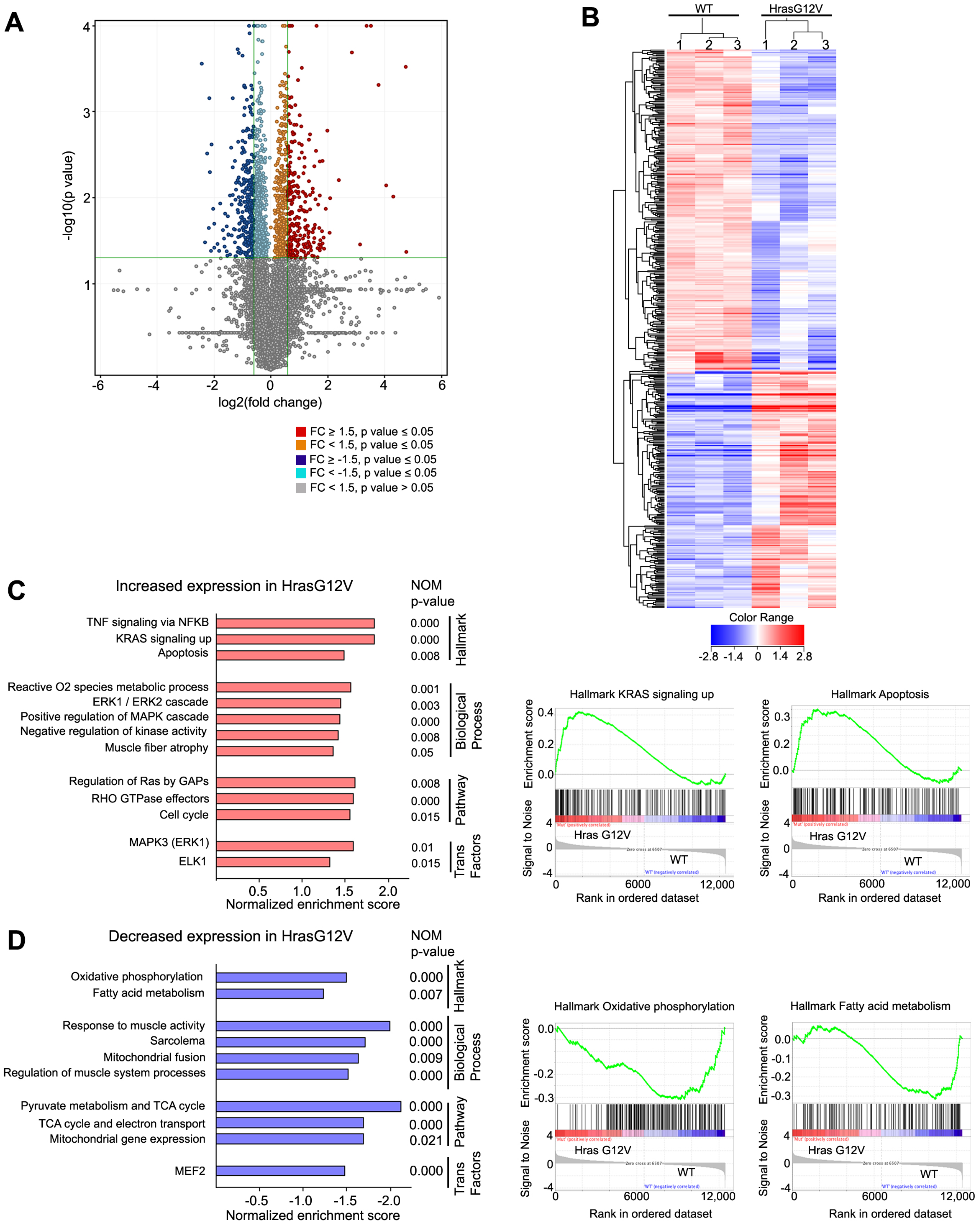

Disease Models & Mechanisms • Supplementary information

Fig. S1. Global alteration in transcription between CS mutant and WT skeletal muscle. (A) Volcano plot of the adjusted p-value (-log p-value) versus log2 fold-change of differentially expressed genes in gastrocnemius muscle from 21-day-old *Hras*^{G12V} and WT. The cut off of +/-1.5 fold-change (vertical green lines) and p-value of 0.05 (horizontal green line) are indicated. Genes with a p-value of >0.05 are shown in gray. Genes with a statistically significant increase in expression (p-value ≤0.05) with a fold-change of ≥1.5 are shown in red and those with <1.5 fold-change are shown in orange. Genes with a statistically significant decrease in expression (p-value ≤0.05) with a fold-change ≥1.5 are shown in blue and those with <1.5 fold-change are shown in light blue. (B) Shown is the heatmap of differentially expressed genes with a fold-change of ≥1.5 and p-value of ≤0.05 by an unpaired t-test (n=3). The three columns on the left of the heatmap indicate WT samples (WT) and the right three columns indicate *Hras*^{G12V} samples. Hierarchical clustering reflecting the similarity of expressed genes and samples are indicated by dendrograms on the left and top of the heatmap, respectively. The pattern of differentially expressed genes clearly distinguishes the *Hras*^{G12V} from WT muscle. Z-scores are used to color the map and indicate the number of standard deviations for the expression level above or below the mean (which is set to zero). Blue indicates negative and red indicates positive relative gene expression. (C, D) Gene Set Enrichment Analysis (GSEA) was performed on the normalized Fragments per Kilobase of transcript per Million mapped reads (FPKM) expression data, using the Molecular Signatures Database (MSigDB) gene sets. The MSigDB gene set collections examined included Hallmark, Curated (canonical pathways), Regulatory (transcription factor targets) and Gene Ontology. GSEA on the FPKM expression data revealed a similar pattern of differential gene expression between Hras^{G12V} and WT when compared to GO and KEGG analyses on differentially expressed genes with a p-value ≤0.05 and

a fold-change of ≥1.5. (C) Relevant gene sets with significantly increased relative expression in the Hras^{G12V} muscle by GSEA are shown. Numerous genes sets are indicative of increased Ras/MAPK pathway activity including KRAS signaling up, ERK1/2 cascade, positive regulation of MAPK cascade, regulation of Ras by GAPs and both ERK1 and ELK1 transcription factor targets. Moreover, gene sets for apoptosis, cell cycle, reactive oxygen species metabolism and muscle fiber atrophy showed increased expression corroborating our GO analysis that was limited to differentially expressed genes with a p-value ≤0.05 and a fold-change of ≥1.5. Representative GSEA plots indicating increased KRAS signaling up and apoptosis in Hras^{G12V} muscle are shown. (D) Relevant gene sets with significantly decreased expression in the Hras^{G12V} muscle by GSEA are shown. Several gene sets revealed decreased mitochondrial function consistent with GO analysis, including, oxidative phosphorylation, mitochondrial fusion, pyruvate/TCA metabolism, TCA/electron transport and mitochondrial gene expression. Likewise, gene sets for fatty acid metabolism, response to muscle activity and muscle processes were also decreased. Targets of the transcription factor MEF2 were decreased which is consistent with our finding that MEF2 showed decreased phosphorylation in Hras^{G12V} muscle. Also shown are representative GSEA plots reflecting significantly decreased oxidative phosphorylation and fatty acid metabolism in the Hras^{G12V} gastrocnemius muscle.

Supplemental Figure 2

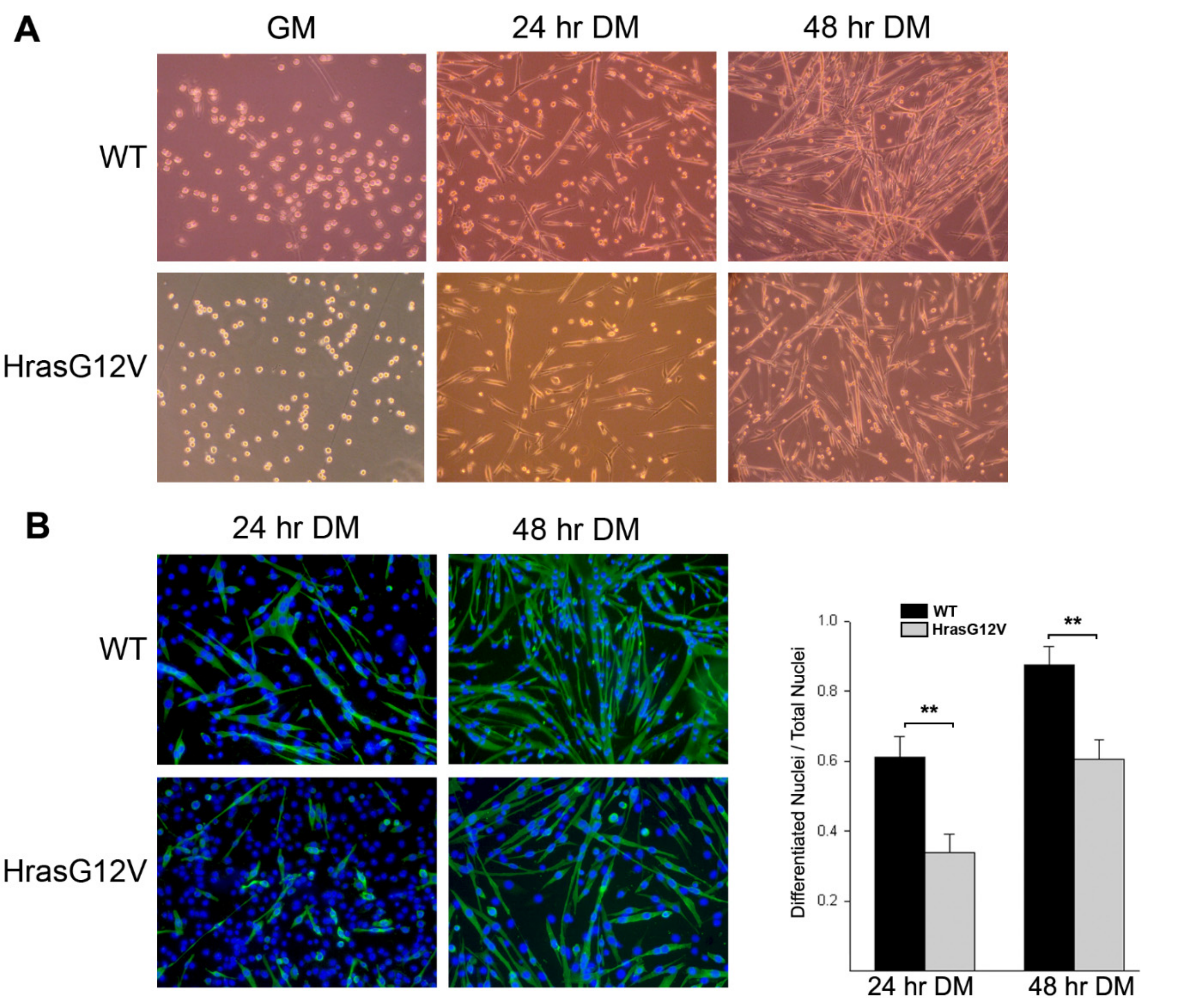

Fig. S2. Primary myoblast cultures from *Hras*^{G12V} and WT mice. (A) Phase contrast images of representative primary myoblasts grown in Growth Media (GM) then switched to Differentiation Media (DM; 10x magnification). Both *Hras*^{G12V} and WT proliferating myoblasts were cultured in GM then switched to low serum DM. At 24 hours in DM, WT myoblasts began to fuse and form myotubes, however *Hras*^{G12V} myoblasts had formed fewer myotubes. At 48 hours in DM, the WT myoblast culture showed extensive myotube formation with elongated, swirling

Disease Models & Mechanisms • Supplementary information

myotubes. In contrast, the *Hras*^{G12V} myoblast cultures demonstrated a stark phenotypic difference with fewer elongated myotubes. (B) Immunofluorescent labeling using an antibody to MyHC distinguished differentiated muscle fibers (MyHC positive, green) and undifferentiated myoblasts which are MyHC negative. Nuclei were counter-stained with DAPI (blue) and images were merged (10x magnification). At 24 and 48 hours in DM, *Hras*^{G12V} myoblast cultures had significantly fewer nuclei in MyHC positive cells compared to WT (n=5; $p < 0.01$). The bars represent the mean +/- SEM, (**) = $p<0.01$.

Supplemental Figure 3

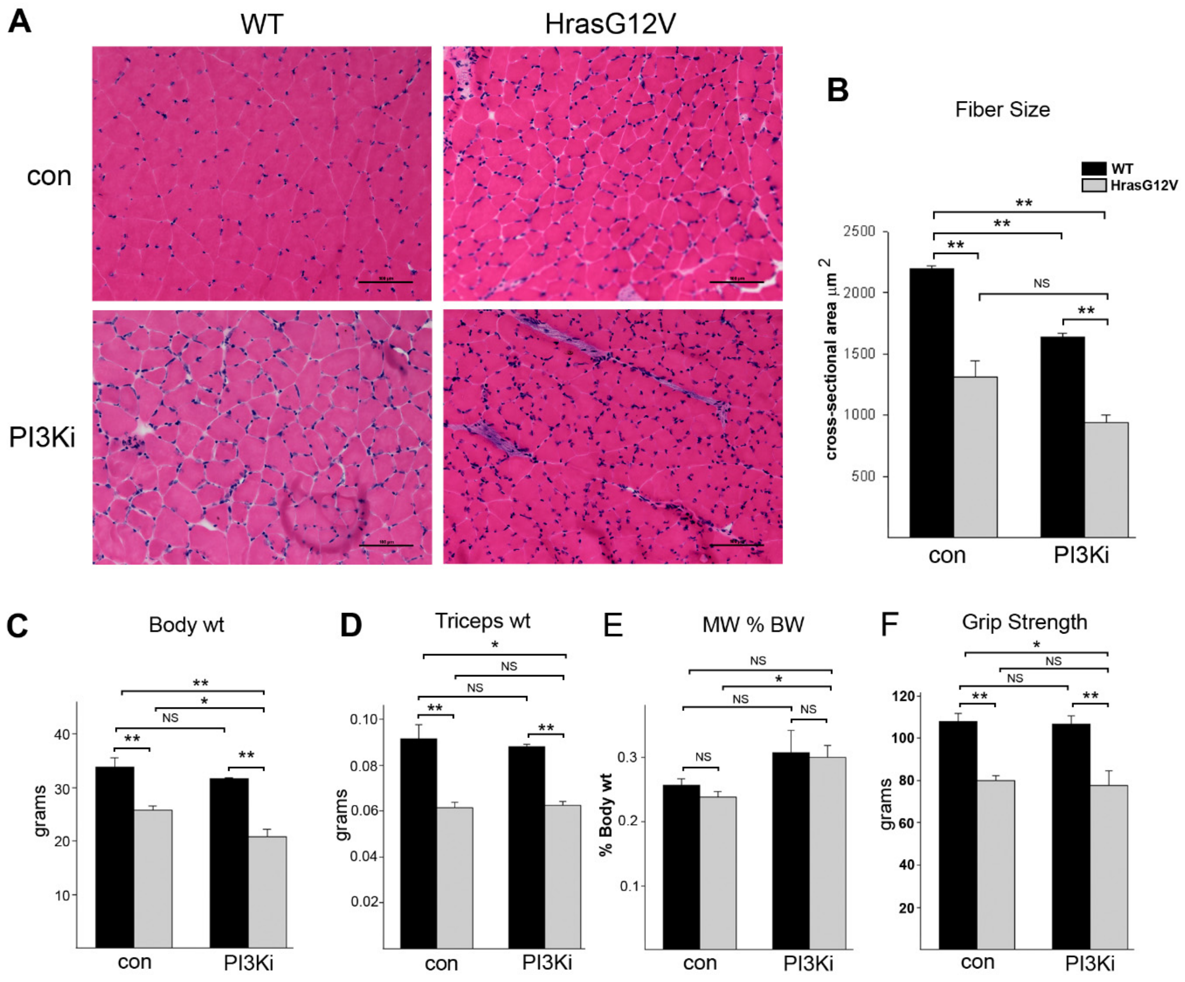

Fig. S3. ***In vivo*** **treatment of** ***Hras***^{G12V} **mice with PI3K inhibitor (PI3Ki) GDC0941.** WT mice and *Hras*^{G12V} were administered either PI3Ki or vehicle, control (con) for 28 days. (A) H&E stained gastrocnemius muscle from adult *Hras*^{G12V} and WT mice treated with PI3Ki or vehicle control (con) showed no phenotypic improvement in the mutant muscle (20x magnification, scale bar = 100 µm). (B) Comparison of gastrocnemius myofiber cross-sectional area between *Hras*^{G12V} and WT mice treated with PI3Ki or vehicle control (con) showed that treatment with PI3Ki resulted in a significant decrease in the *Hras*^{G12V} gastrocnemius muscle fiber

Disease Models & Mechanisms • Supplementary information

cross-sectional area compared to both WT con and WT PI3Ki treated mice (n=3, p<0.01). There was also a significant decrease in muscle fiber cross-sectional area between the WT PI3Ki treated compared to WT untreated (con) mice (n=3, p<0.01). Comparison of (C) body weight, (D) dissected triceps muscle weight, and (F) fore limb grip strength between PI3Ki treated and untreated WT and *Hras*^{G12V} control (con) mice also showed no improvement in the myopathic phenotype in the mutant mice after treatment. (C) Treatment with PI3Ki resulted in a significant decrease in body weight of both WT (n=3, p<0.01) and *Hras*^{G12V} (n=3, p<0.05) mice compared to vehicle treated (con) mice. (D) There was no significant difference in triceps weight between *Hras*^{G12V} treated and *Hras*^{G12V} untreated (con) mice (n=3, p=0.81). (E) The percentage of total body weight of the triceps muscle (MW % BW) showed an increase in the *Hras*^{G12V} mice compared to the untreated *Hras*^{G12V} mice probably resulting from the overall decrease in body weight in the treated mice (n=3, P<0.05). (F) There was no significant difference in grip strength between *Hras*^{G12V} treated and *Hras*^{G12V} untreated (con) mice (n=3, p=0.67). The bars represent the mean +/- SEM, (*) = p<0.05, (**) = p<0.01, (NS) not significant.

Table S1. The 207 relatively increased gene transcripts which have a positive fold change greater than 1.5 (FC > 1.5) and p-value less than or equal to 0.05 ($p \leq 0.05$) for *Hras*^{G12V} / WT (n=3).

Gene ID	Gene Description	Fold Change HrasG12V/WT	p value
Mir7079		221.05	0.000334
Mir678		145.95	3.33E-09
Kcne1l	potassium voltage-gated channel, Isk-related family, member 1-like	27.38	0.000781
Snord16a	small nucleolar RNA, C/D box 16A	27.02	0.043765
S100a8	S100 calcium binding protein A8 (calgranulin A)	18.37	0.007724
S100a9	S100 calcium binding protein A9 (calgranulin B)	15.57	0.004774
Mir3473e		13.19	0.000282
4930544D05Rik		11.62	0.000874
Chrne	cholinergic receptor, nicotinic, epsilon polypeptide	10.29	0.000798
Ucp1	uncoupling protein 1 (mitochondrial, proton carrier)	8.49	0.029967
Chrna1	cholinergic receptor, nicotinic, alpha polypeptide 1 (muscle)	7.06	0.000267
Mt3	metallothionein 3	5.43	0.010184
Dok3	docking protein 3	4.17	0.016459
Slfn2	schlafen 2	4.17	0.040488
Colq	similar to Collagen-like tail subunit (single strand of homotrimer) of asymmetric acetylcholinesterase;	4.01	0.006302
Mpv17l	Mpv17 transgene, kidney disease mutant-like	3.80	0.00257
Snhg9	small nucleolar RNA host gene (non-protein coding) 9	3.74	0.024745
Irf8	interferon regulatory factor 8	3.71	0.01811
Ifitm1	interferon induced transmembrane protein 1	3.36	0.031706
Limd2	similar to epithelial protein lost in neoplasm; LIM domain containing 2	3.30	0.030833
Dbp	D site albumin promoter binding protein	3.30	0.036976
Ccl8	chemokine (C-C motif) ligand 8	3.30	0.021719
Tbc1d10c	TBC1 domain family, member 10c	3.24	0.045997
Akap11	A kinase (PRKA) anchor protein 11	3.21	0.001941
Hs6st2	heparin sulfate 6-O-sulfotransferase 2	3.11	0.010536
Traf3ip3	TRAF3 interacting protein 3	3.03	0.039998
Etv5	ets variant gene 5	3.01	0.001568
Cpne2	copine II	3.00	0.002465
Tbx21	T-box 21	2.99	0.019037
H2-K1	histocompatibility 2, K1, K region; similar to H-2K(d) antigen	2.97	0.023674
Mfsd7a	major facilitator superfamily domain containing 7A	2.93	0.003322
Cotl1	coactosin-like 1 (Dictyostelium)	2.93	0.036868
Apoc1	apolipoprotein C-I	2.90	0.023097
Ifi47	interferon gamma inducible protein 47	2.89	0.044425

Disease Models & Mechanisms • Supplementary information

Psmb8	proteasome (prosome, macropain) subunit, beta type 8	2.88	0.009197
Ache	acetylcholinesterase	2.87	0.012405
Spi1	Transcription factor PU.1 is a protein that in humans is encoded by the SPI1 gene	2.80	0.021006
Pik3cd	phosphatidylinositol 3-kinase catalytic delta polypeptide; RIKEN cDNA 2610208K16 gene	2.75	0.03479
Mir703		2.69	0.007081
Arhgdib	Rho, GDP dissociation inhibitor (GDI) beta	2.68	0.049863
E330011O21Rik	RIKEN cDNA E330011O21 gene	2.66	0.027812
H2-M3	histocompatibility 2, M region locus 3	2.62	0.004227
Snca	synuclein, alpha	2.61	0.014611
Tnfaip8l2	tumor necrosis factor, alpha-induced protein 8-like 2	2.60	0.013651
Gpsm3	G-protein signalling modulator 3 (AGS3-like, C. elegans)	2.60	0.01897
Fam65b	family with sequence similarity 65, member B	2.60	0.045367
Msc	musculin	2.58	0.019707
Ufsp1	UFM1-specific peptidase 1	2.56	0.047789
Fam178b	family with sequence similarity 178, member B	2.55	0.026775
Ncf4	neutrophil cytosolic factor 4	2.55	0.021508
Rab13	RAB13, member RAS oncogene family	2.52	0.020791
Fzd10	frizzled homolog 10 (Drosophila)	2.51	0.004205
Lst1	leukocyte specific transcript 1	2.51	0.048566
Marcksl1	MARCKS-like 1; predicted gene 9106	2.48	0.037374
Il17ra	interleukin 17 receptor A	2.44	0.026974
Cdc25b	cell division cycle 25 homolog B	2.41	0.028307
Hcls1	hematopoietic cell specific Lyn substrate 1	2.40	0.034175
Tyrobp	TYRO protein tyrosine kinase binding protein	2.39	0.011707
Ltc4s	leukotriene C4 synthase	2.38	0.048743
Col23a1	similar to procollagen, type XXIII, alpha 1; collagen, type XXIII, alpha 1	2.37	0.0049
Mcm3	minichromosome maintenance deficient 3 (S. cerevisiae)	2.36	0.015886
Gfra4	glial cell line derived neurotrophic factor family receptor alpha 4	2.34	0.048338
Plvap	plasmalemma vesicle associated protein	2.33	0.02535
Slc9a3r1	solute carrier family 9 (sodium/hydrogen exchanger), member 3 regulator 1	2.33	0.028559
Gstt3	glutathione S-transferase, theta 3	2.31	0.029384
Acat2	acetyl-Coenzyme A acetyltransferase 2	2.30	0.010742
Apoe	apolipoprotein E	2.29	0.002821
Hmgb2	predicted gene 13160; predicted gene 8681	2.29	0.046673
Rogdi	rogdi homolog (Drosophila)	2.27	0.004668
Clcf1	cardiotrophin-like cytokine factor 1	2.26	0.03415
Ptpn18	protein tyrosine phosphatase, non-receptor type 18	2.25	0.006415
Rasa3	RAS p21 protein activator 3	2.24	0.007453

Disease Models & Mechanisms • Supplementary information

5730408K05Rik		2.24	0.02412
Tmsb10	predicted gene 3787; predicted gene 9844; predicted gene 8034; similar to thymosin, beta 10; thymosin, beta 10	2.23	0.001226
Ccdc36	coiled-coil domain containing 36	2.21	0.038904
Gimap4	GTPase, IMAP family member 4	2.20	0.00575
Relb	avian reticuloendotheliosis viral (v-rel) oncogene related B	2.19	0.003198
Spata24	RIKEN cDNA 5133400G04 gene	2.15	0.028307
Cacnb3	calcium channel, voltage-dependent, beta 3 subunit	2.15	0.003283
Map3k7cl	mitogen acitvated protein kinase kinase kinase 7, C-terminal like	2.15	0.035981
Tmco4	transmembrane and coiled-coil domains 4	2.13	0.010195
Sh3bp1	similar to SH3-domain binding protein 1; SH3-domain binding protein 1; similar to SH3 domain-binding protein 1 (3BP-1)	2.13	0.000741
Trp63	transformation related protein 63	2.12	0.014566
Erdr1	erythroid differentiation regulator 1	2.08	0.020565
H2-T9	histocompatibility 2, T region locus 9	2.06	0.03342
Lyl1	lymphoblastomic leukemia 1	2.06	0.043809
Nog	noggin	2.01	0.024273
Gm13889	predicted gene 13889	2.01	0.026431
Ube2c	ubiquitin-conjugating enzyme E2C; predicted gene 8956	2.01	0.034028
Snhg7	small nucleolar RNA host gene (non-protein coding) 7	2.00	0.034631
Gstt1	glutathione S-transferase, theta 1	1.99	0.008087
Gpx3	glutathione peroxidase 3	1.97	0.01809
Rps6ka1	ribosomal protein S6 kinase polypeptide 1	1.97	0.00658
Gm11974	predicted gene 11974	1.96	0.002788
Mob3a	MOB kinase activator 3A	1.96	0.017409
Cd72	CD72 antigen	1.96	0.000262
Rab26os	RAB26, member RAS oncogene family opposite strand	1.94	0.005001
Maged2	similar to melanoma antigen family D, 2; melanoma antigen, family D, 2	1.94	0.000151
Srebf1	sterol regulatory element binding transcription factor 1	1.93	0.002719
Ptk7	PTK7 protein tyrosine kinase 7	1.93	0.006789
Pla2g7	phospholipase A2, group VII (platelet-activating factor acetylhydrolase, plasma)	1.93	0.036468
Myl4	myosin, light polypeptide 4	1.91	0.030823
Rbm3	predicted gene 15453; RNA binding motif protein 3	1.91	0.018098
Socs1	suppressor of cytokine signaling 1	1.90	0.001665
Bst2	bone marrow stromal cell antigen 2	1.90	0.044713
Tuba1b	predicted gene 3756; tubulin, alpha 1B; predicted gene 5620; similar to alpha-tubulin isotype M-alpha-2; 14150; 3226	1.89	0.035198
4930404N11Rik		1.88	0.016761
Gimap1	GTPase, IMAP family member 1	1.87	0.028032
Ier5	immediate early response 5	1.87	0.044592
Smc4	structural maintenance of chromosomes 4	1.86	0.049582

Mapk11	mitogen-activated protein kinase 11	1.86	0.016633
Ccdc34	coiled-coil domain containing 34	1.86	0.025634
H2-D1	histocompatibility 2, D region; histocompatibility 2, D region locus 1	1.86	0.024666
Fxyd5	FXYD domain-containing ion transport regulator 5	1.85	0.000999
Npl	N-acetylneuraminate pyruvate lyase	1.85	0.041963
Mcm2	minichromosome maintenance deficient 2 mitotin (S. cerevisiae)	1.85	0.008712
Cyba	cytochrome b-245, alpha polypeptide	1.85	0.011141
Sh2b2	SH2B adaptor protein 2	1.85	0.008221
Galns	galactosamine (N-acetyl)-6-sulfate sulfatase	1.85	0.0084
Capg	capping protein (actin filament), gelsolin-like	1.84	0.003496
Mir17hg		1.84	0.039948
Cib1	calcium and integrin binding 1 (calmyrin)	1.83	0.004413
Rgs10	regulator of G-protein signalling 10	1.81	0.006035
Psmb10	proteasome (prosome, macropain) subunit, beta type 10	1.81	0.024674
Pafah1b3	platelet-activating factor acetylhydrolase, isoform 1b, subunit 3	1.81	0.033246
Mag	myelin-associated glycoprotein	1.81	0.0255
Tspan15	tetraspanin 15	1.80	0.004916
upf3b	reproductive homeobox 3B; upF3 regulator of nonsense transcripts homolog B (yeast); reproductive homeobox 1; NFKB activating protein	1.78	0.046731
B2m	beta-2 microglobulin	1.77	0.027258
BC033916	cDNA sequence BC017643	1.77	0.024492
Rpl3	predicted gene 5879; similar to 60S ribosomal protein L3 (J1 protein);	1.77	0.008048
Gpx1	glutathione peroxidase 1	1.76	0.04801
Col13a1	collagen, type XIII, alpha 1	1.76	0.008295
Clec2d	C-type lectin domain family 2, member d	1.76	0.020611
Nt5c	5',3'-nucleotidase, cytosolic	1.75	0.032779
Bok	BCL2-related ovarian killer protein	1.75	0.002243
A930005H10Rik		1.75	0.047578
Lbh	limb-bud and heart	1.75	0.031924
1700037H04Rik		1.74	0.038847
Cachd1	cache domain containing 1; similar to Cache domain containing 1	1.73	0.046085
AI414108		1.73	0.042631
Ncam1	neural cell adhesion molecule 1	1.73	0.009961
Tmem176b	transmembrane protein 176B	1.72	0.027821
Unc93b1	unc-93 homolog B1 (C. elegans)	1.70	0.039756
Fen1	flap structure specific endonuclease 1	1.70	0.005525
Dcaf15	cDNA sequence BC057552	1.70	0.006979
Rfc3	replication factor C (activator 1) 3	1.70	0.043348
Tubb5	tubulin, beta 5	1.70	0.010736
Golm1	golgi membrane protein 1	1.69	0.016933

Nfkb2	nuclear factor of kappa light polypeptide gene enhancer in B-cells 2	1.69	0.005978
Taf1d	TATA box binding protein (Tbp)-associated factor, RNA polymerase I, D; predicted gene 13487	1.69	0.000647
Hsd11b1	hydroxysteroid 11-beta dehydrogenase 1	1.68	0.029646
Eid1	EP300 interacting inhibitor of differentiation 1	1.67	0.008622
Hyi	hydroxypyruvate isomerase homolog (E. coli)	1.67	0.038104
Fam117a	family with sequence similarity 117, memberA	1.67	0.032035
Cfl1	cofilin 1, non-muscle; similar to Cofilin-1 (Cofilin, non-muscle isoform); predicted gene 6180	1.67	0.042145
Il18bp	interleukin 18 binding protein	1.66	0.008604
Grap	GRB2-related adaptor protein	1.66	0.011738
Atp8a2	ATPase, aminophospholipid transporter-like, class I, type 8A, member 2	1.66	0.024178
Crip3	cysteine-rich protein 3	1.65	0.012329
Gimap6	GTPase, IMAP family member 6	1.65	0.00032
Rpl13a	similar to 60S ribosomal protein L13a (Transplantation antigen P198)	1.65	0.041363
Bbc3	cDNA sequence BC037034	1.65	0.043495
Tcirg1	T-cell, immune regulator 1, ATPase, H+ transporting, lysosomal V0 protein A3	1.65	0.008747
Tmsb4x	thymosin, beta 4, X chromosome; similar to thymosin beta-4	1.65	0.013281
Cxcr2	interleukin 8 receptor, beta	1.64	0.016122
Tfpi	tissue factor pathway inhibitor	1.64	0.012027
Traf5	TNF receptor-associated factor 5	1.63	0.040085
Tap2	transporter 2, ATP-binding cassette, sub-family B (MDR/TAP)	1.63	0.019417
Anp32b	acidic (leucine-rich) nuclear phosphoprotein 32 family, member B	1.63	0.032314
1700071M16Rik		1.63	0.047057
Ezh2	enhancer of zeste homolog 2 (Drosophila)	1.63	0.025318
Nmi	N-myc (and STAT) interactor	1.62	0.011887
Flt3l	FMS-like tyrosine kinase 3 ligand	1.62	0.034141
Ppan	peter pan homolog (Drosophila)	1.61	0.013503
1500011B03Rik		1.61	0.016156
Trim12c	tripartite motif-containing 12C	1.61	0.012067
Cd82	CD82 antigen	1.61	0.013783
Sh3bgrl3	SH3 domain binding glutamic acid-rich protein-like 3	1.61	0.005733
Prpsap1	phosphoribosyl pyrophosphate synthetase-associated protein 1	1.61	0.024971
Nek6	NIMA (never in mitosis gene a)-related expressed kinase 6	1.60	0.013275
H2afj	H2A histone family, member J	1.60	0.037853
Ptma	predicted gene 12504; 9800; 4617; 6625; 7614; similar to prothymosin alpha;	1.60	0.001358
Mcm7	minichromosome maintenance deficient 7 (S. cerevisiae)	1.60	0.007355
Tmem51	transmembrane protein 51	1.60	0.001189
BC037034	cDNA sequence BC033916	1.59	0.007169
Clic1	chloride intracellular channel 1	1.58	0.012901

BC017643	BCL2 binding component 3	1.58	0.033734
Tnfrsf23	tumor necrosis factor receptor superfamily, member 23	1.57	0.004846
Orc6	origin recognition complex subunit 6	1.56	0.025358
Sltm	SAFB-like, transcription modulator	1.56	0.021361
Ngfrap1	nerve growth factor receptor (TNFRSF16) associated protein 1	1.55	0.033783
Crlf2	cytokine receptor-like factor 2	1.55	0.021905
2610002J02Rik		1.55	0.000797
Sirt7	sirtuin 7 (silent mating type information regulation 2, homolog) 7 (S. cerevisiae)	1.55	0.002344
Pax7	paired box gene 7	1.55	0.006093
Polr1a	polymerase (RNA) I polypeptide A	1.54	0.048046
Lrp4	low density lipoprotein receptor-related protein 4	1.54	0.033786
Trp53	transformation related protein 53	1.53	0.005003
Aldh1l1	similar to Aldehyde dehydrogenase 1 family, member L1	1.53	0.010951
Pabpn1	poly(A) binding protein, nuclear 1	1.53	0.041722
Emp3	epithelial membrane protein 3	1.52	0.049967
Snrnp70	small nuclear ribonucleoprotein 70 (U1)	1.52	0.047043
Nop56	predicted gene 5243; NOP56 ribonucleoprotein homolog (yeast)	1.51	0.030118
Pold1	polymerase (DNA directed), delta 1, catalytic subunit	1.51	0.029542
Ralgds	ral guanine nucleotide dissociation stimulator	1.50	0.012773
Pvrl2	poliovirus receptor-related 2	1.50	0.045034

Disease Models & Mechanisms • Supplementary information

Table S2. The 280 relatively decreased gene transcripts which have a positive fold change greater than 1.5 (FC > 1.5) and p-value less than or equal to 0.05 ($p \leq 0.05$) for *Hras*^{G12V} / WT (n=3).

Gene ID	Gene Description	Fold Change HrasG12V/WT	p value
Nos1	nitric oxide synthase 1, neuronal	-5.14	0.00085
Mylk4	myosin light chain kinase family, member 4	-4.90	0.00139
Kera	keratocan	-4.71	0.01623
Wif1	Wnt inhibitory factor 1	-4.64	0.04209
Mir8104		-4.49	0.00069
Ucp3	uncoupling protein 3 (mitochondrial, proton carrier)	-4.41	0.01047
1500015O10Rik		-4.34	0.04655
Pdk4	pyruvate dehydrogenase kinase, isoenzyme 4	-4.19	0.03144
Best3	bestrophin 3	-4.16	0.00155
Prima1	proline rich membrane anchor 1	-3.90	0.02084
Mir6403		-3.90	0.02240
Aqp4	aquaporin 4	-3.75	0.02515
Tnmd	tenomodulin	-3.70	0.04803
Comp	cartilage oligomeric matrix protein	-3.63	0.04895
Cpxm2	carboxypeptidase X 2 (M14 family)	-3.50	0.01810
Col11a2	collagen, type XI, alpha 2	-3.27	0.02905
Cilp2	cartilage intermediate layer protein 2	-3.07	0.03854
Gm13031	predicted gene 13031	-2.96	0.01128
Padi2	peptidyl arginine deiminase, type II; similar to peptidyl arginine deiminase, type II	-2.84	0.04401
Apoo-ps	apolipoprotein O, pseudogene	-2.84	0.03082
Col8a2	collagen, type VIII, alpha 2	-2.70	0.03322
Ptpn3	protein tyrosine phosphatase, non-receptor type 3	-2.68	0.00930
Tmem56	transmembrane protein 56	-2.68	0.00841
Slc40a1	solute carrier family 40 (iron-regulated transporter), member 1	-2.66	0.00884
Tgoln2	trans-golgi network protein 2; trans-golgi network protein	-2.65	0.03964
Gbp10	predicted gene, EG634650; guanylate-binding protein 10	-2.61	0.04636
Hmga2-ps1	high mobility group AT-hook 2, pseudogene 1	-2.60	0.00411
Phka1	phosphorylase kinase alpha 1	-2.60	0.00071
Tmem8c	transmembrane protein 8C, myomaker, myoblast fusion factor	-2.56	0.03241
Gdap1	ganglioside-induced differentiation-associated-protein 1	-2.49	0.00394
Hipk2	homeodomain interacting protein kinase 2	-2.46	0.00692
2310016D03Rik		-2.44	0.00034
Ptx4	Pentraxin 4, paralogue NPTX2	-2.37	0.03359
Tm6sf1	transmembrane 6 superfamily member 1	-2.36	0.01161

Disease Models & Mechanisms • Supplementary information

Kcnc1	potassium voltage gated channel, Shaw-related subfamily, member 1	-2.36	0.03970
Asb15	ankyrin repeat and SOCS box-containing 15	-2.34	0.00551
Tspan8	tetraspanin 8	-2.32	0.03640
Slc25a25	solute carrier family 25 (mitochondrial carrier, phosphate carrier)	-2.30	0.01543
Pon3	paraoxonase 3	-2.26	0.00583
Ccdc85c	predicted gene 9010	-2.24	0.03976
Cdc14a	similar to Dual specificity protein phosphatase CDC14A (CDC14 cell division cycle 14 homolog A);	-2.22	0.00182
Pank1	pantothenate kinase 1	-2.20	0.03194
Acot11	acyl-CoA thioesterase 11	-2.18	0.02588
Ankrd33b	ankyrin repeat domain 33B	-2.17	0.00729
Sorbs2	sorbin and SH3 domain containing 2	-2.17	0.03937
Fam179a	family with sequence similarity 179, member A	-2.16	0.03206
Slc25a22	solute carrier family 25 (mitochondrial carrier, glutamate), member 22	-2.16	0.02459
Nfil3	similar to NFIL3/E4BP4 transcription factor; nuclear factor, interleukin 3, regulated	-2.15	0.04127
Prkg1	protein kinase, cGMP-dependent, type I	-2.14	0.01375
Rgs5	regulator of G-protein signaling 5	-2.13	0.02734
Kcnf1	potassium voltage-gated channel, subfamily F, member 1	-2.12	0.01150
Ptgis	prostaglandin I2 (prostacyclin) synthase	-2.11	0.03941
1700018L02Rik		-2.08	0.00204
Cacng7	calcium channel, voltage-dependent, gamma subunit 7	-2.05	0.02866
Hecw2	HECT, C2 and WW domain containing E3 ubiquitin protein ligase 2	-2.05	0.00064
Vwa3a	von Willebrand factor A domain containing 3A	-2.04	0.02119
Ctnna3	catenin (cadherin associated protein), alpha 3	-2.03	0.00616
Ell2	elongation factor RNA polymerase II 2	-2.03	0.00328
Pgm2l1	phosphoglucomutase 2-like 1	-2.02	0.00800
Dach1	dachshund 1 (Drosophila)	-2.01	0.04806
Mstn	myostatin	-2.01	0.04974
Mcf2l	mcf.2 transforming sequence-like	-2.01	0.00428
Adamts9	a disintegrin-like and metallopeptidase (reprolysin type) with thrombospondin type 1 motif, 9	-2.01	0.01203
Ednrb	endothelin receptor type B	-2.01	0.01818
St3gal6	ST3 beta-galactoside alpha-2,3-sialyltransferase 6	-1.97	0.00389
Nt5c1a	5'-nucleotidase, cytosolic IA	-1.97	0.00807
Col22a1	collagen, type XXII, alpha 1	-1.95	0.02450
Asb18	ankyrin repeat and SOCS box-containing 18	-1.95	0.01996
Ppp1r3c	protein phosphatase 1, regulatory (inhibitor) subunit 3C	-1.95	0.03836
Pptc7	PTC7 protein phosphatase homolog (S. cerevisiae)	-1.95	0.00854
Fitm2	fat storage-inducing transmembrane protein 2	-1.94	0.00251
Rap1gap	Rap1 GTPase-activating protein	-1.94	0.00876

4930578C19Rik		-1.94	0.04158
Tecpr2	tectonin beta-propeller repeat containing 2	-1.93	0.02351
Mafb	v-maf musculoaponeurotic fibrosarcoma oncogene family, protein B (avian)	-1.92	0.00600
Ccdc85a	coiled-coil domain containing 85A	-1.92	0.00648
Nceh1	arylacetamide deacetylase-like 1	-1.91	0.01619
Gas2	growth arrest specific 2	-1.91	0.00423
Slc38a4	solute carrier family 38, member 4	-1.91	0.00475
Gm20319	predicted gene, 20319	-1.91	0.04914
9430020K01Rik		-1.90	0.01864
Flt1	FMS-like tyrosine kinase 1	-1.89	0.00210
Parm1	RIKEN cDNA 9130213B05 gene	-1.89	0.01903
Prkca	protein kinase C, alpha	-1.89	0.00666
Fgd4	FYVE, RhoGEF and PH domain containing 4	-1.89	0.02808
Rps6ka2	ribosomal protein S6 kinase, polypeptide 2; similar to Ribosomal protein S6 kinase, polypeptide 2	-1.88	0.02845
Enpp4	ectonucleotide pyrophosphatase/phosphodiesterase 4	-1.88	0.01209
Pex11a	peroxisomal biogenesis factor 11 alpha	-1.88	0.02524
Prom1	prominin 1	-1.88	0.01073
Sfxn5	sideroflexin 5; hypothetical protein LOC100044130	-1.87	0.00035
Gm6307	predicted gene 6307	-1.87	0.04979
Ppm1l	protein phosphatase 1 (formerly 2C)-like	-1.87	0.02422
Zfp770	zinc finger protein 770	-1.85	0.01103
Zbtb16	zinc finger and BTB domain containing 16	-1.84	0.00164
Gpr116	G protein-coupled receptor 116	-1.84	0.03422
Mpdz	multiple PDZ domain protein	-1.84	0.00610
Cyp2u1	cytochrome P450, family 2, subfamily u, polypeptide 1	-1.83	0.01106
Abcc4	ATP-binding cassette, sub-family C (CFTR/MRP), member 4	-1.83	0.00225
Pde7b	phosphodiesterase 7B	-1.83	0.00583
Hnmt	histamine N-methyltransferase	-1.83	0.00040
Gpr17	G protein-coupled receptor 17	-1.83	0.00507
Homer1	homer homolog 1 (Drosophila)	-1.83	0.00194
Acvr1b	activin A receptor, type 1B	-1.83	0.03622
Sox7	SRY-box containing gene 7	-1.82	0.00081
Npas2	neuronal PAS domain protein 2	-1.81	0.03190
Sybu	syntabulin (syntaxin-interacting)	-1.81	0.00231
Bcl6	B-cell leukemia/lymphoma 6	-1.80	0.03371
Nipal3	NIPA-like domain containing 3; similar to NIPA-like domain containing 3	-1.80	0.00639
Mrgprh	MAS-related GPR, member H	-1.80	0.02550
9430037G07Rik		-1.79	0.04668
Dnajc27	DnaJ (Hsp40) homolog, subfamily C, member 27	-1.79	0.00591

Wwp1	predicted gene 13416; WW domain containing E3 ubiquitin protein ligase 1	-1.79	0.00740
G630090E17Rik	predicted gene, 100041085; similar to Na+ dependent glucose transporter 1	-1.78	0.03753
Lin7a	lin-7 homolog A (C. elegans)	-1.78	0.00368
Tmem65	transmembrane protein 65	-1.78	0.02928
Fgfr3	fibroblast growth factor receptor 3	-1.78	0.02019
P2ry1	purinergic receptor P2Y, G-protein coupled 1	-1.77	0.01512
Fam120c	family with sequence similarity 120, member C	-1.77	0.02469
Cyb5d2	cytochrome b5 domain containing 2	-1.76	0.01321
Ldlrad4	low density lipoprotein receptor class A domain containing 4	-1.76	0.00884
Hspa4l	heat shock protein 4 like	-1.76	0.02195
Tbc1d1	TBC1 domain family, member 1; similar to TBC1 domain family member 1	-1.76	0.04750
Slc19a2	solute carrier family 19 (thiamine transporter), member 2	-1.76	0.00179
Parvb	parvin, beta; similar to parvin, beta	-1.75	0.03705
Fut10	fucosyltransferase 10	-1.75	0.00515
Jam2	junction adhesion molecule 2	-1.74	0.00003
Kcnma1	potassium large conductance calcium-activated channel, subfamily M, alpha member 1	-1.74	0.01485
Abcb7	ATP-binding cassette, sub-family B (MDR/TAP), member 7	-1.73	0.04239
Eltd1	EGF, latrophilin seven transmembrane domain containing 1	-1.73	0.01958
Styx	serine/threonine/tyrosine interaction protein; predicted gene 14698	-1.73	0.02384
Cdnf	cerebral dopamine neurotrophic factor	-1.71	0.01385
Rasgrf2	RAS protein-specific guanine nucleotide-releasing factor 2	-1.71	0.03503
Egf	epidermal growth factor	-1.71	0.00008
Ibtk	inhibitor of Bruton agammaglobulinemia tyrosine kinase	-1.71	0.04762
Slc2a12	solute carrier family 2 (facilitated glucose transporter), member 12	-1.71	0.01478
9330159F19Rik		-1.70	0.04093
Ptprb	protein tyrosine phosphatase, receptor type, B	-1.70	0.03955
Phkb	phosphorylase kinase beta	-1.70	0.02384
Zfp189	zinc finger protein 189	-1.69	0.02486
Tmem245	transmembrane protein 245	-1.69	0.02339
Abcb1a	ATP-binding cassette, sub-family B (MDR/TAP), member 1A	-1.69	0.01267
Ppargc1a	peroxisome proliferative activated receptor, gamma, coactivator 1 alpha	-1.69	0.00077
Ablim3	actin binding LIM protein family, member 3	-1.69	0.00412
Mlxip	MLX interacting protein	-1.68	0.00696
Myadm	myeloid-associated differentiation marker	-1.68	0.01306
Pnpla8	patatin-like phospholipase domain containing 8	-1.68	0.01947
Dbndd1	dysbindin (dystrobrevin binding protein 1) domain containing 1	-1.68	0.04503
Abcd3	ATP-binding cassette, sub-family D (ALD), member 3	-1.67	0.01619
Tmem64	transmembrane protein 64	-1.67	0.00130
6530402F18Rik		-1.67	0.02011

Disease Models & Mechanisms • Supplementary information

Atp2b3	ATPase, Ca++ transporting, plasma membrane 3	-1.67	0.01697
Daam2	dishevelled associated activator of morphogenesis 2	-1.67	0.02221
Pigg	phosphatidylinositol glycan anchor biosynthesis, class G	-1.67	0.02749
Hr	hairless	-1.66	0.00322
Spata1	spermatogenesis associated 1	-1.66	0.03797
Aig1	androgen-induced 1	-1.66	0.00908
C2cd2	C2 calcium-dependent domain containing 2	-1.66	0.03036
Gpr56	G protein-coupled receptor 56	-1.66	0.01556
Pcnx	pecanex homolog (Drosophila)	-1.66	0.00004
Uggt2	UDP-glucose ceramide glucosyltransferase-like 2	-1.65	0.02337
Acad11	nephronophthisis 3 (adolescent); acyl-Coenzyme A dehydrogenase family, member 11	-1.65	0.01664
Pkp4	plakophilin 4	-1.65	0.04181
Epha4	Eph receptor A4	-1.65	0.00563
Fbxl17	F-box and leucine-rich repeat protein 17	-1.65	0.00554
Gid4	GID Complex Subunit 4, Vacuolar Import And Degradation Protein 24 Homolog	-1.65	0.00351
Kbtbd12	kelch domain containing 6	-1.64	0.04767
Col4a1	collagen, type IV, alpha 1	-1.64	0.02249
Lypla1	lysophospholipase 1	-1.64	0.00714
Lhx6	LIM homeobox protein 6	-1.64	0.01431
Mrs2	MRS2 magnesium homeostasis factor homolog (S. cerevisiae)	-1.64	0.01283
St6galnac2	ST6 (alpha-N-acetyl-neuraminyl-2,3-beta-galactosyl-1,3)-N-acetylgalactosaminide alpha-2,6-sialyltransferase 2	-1.64	0.00881
Dlat	dihydrolipoamide S-acetyltransferase (E2 component of pyruvate dehydrogenase complex)	-1.63	0.04336
Btbd3	BTB (POZ) domain containing 3	-1.63	0.03839
Atxn1	ataxin 1	-1.63	0.02188
Nt5dc3	5'-nucleotidase domain containing 3	-1.63	0.03372
Raver2	ribonucleoprotein, PTB-binding 2	-1.63	0.04525
Mast4	microtubule associated serine/threonine kinase family member 4	-1.63	0.02113
B4galt4	UDP-Gal:betaGlcNAc beta 1,4-galactosyltransferase, polypeptide 4	-1.63	0.00237
Lgr4	leucine-rich repeat-containing G protein-coupled receptor 4	-1.62	0.02028
Jmjd4	jumonji domain containing 4	-1.62	0.02433
Ank	progressive ankylosis	-1.62	0.04949
Aqp7	aquaporin 7	-1.62	0.01863
Agtpbp1	ATP/GTP binding protein 1	-1.62	0.00102
Slc1a1	solute carrier family 1 (neuronal/epithelial high affinity glutamate transporter, system Xag), member 1	-1.62	0.02114
Map3k6	mitogen-activated protein kinase kinase kinase 6	-1.62	0.02757
Hoxa3	homeo box A3	-1.62	0.04080
Lrrc36	leucine rich repeat containing 36	-1.62	0.04506

Disease Models & Mechanisms • Supplementary information

Mtfr1	mitochondrial fission regulator 1	-1.61	0.01153
Prkcg	protein kinase C, gamma	-1.61	0.02975
Trp53i11	transformation related protein 53 inducible protein 11	-1.61	0.00640
Nova2	neuro-oncological ventral antigen 2	-1.61	0.00612
Ston2	stonin 2	-1.61	0.03365
Xirp2	xin actin-binding repeat containing 2	-1.61	0.01964
Uhrf1bp1	UHRF1 (ICBP90) binding protein 1	-1.61	0.00052
Tob1	transducer of ErbB-2.1	-1.61	0.00452
Ugp2	UDP-glucose pyrophosphorylase 2	-1.60	0.02812
Acsl1	acyl-CoA synthetase long-chain family member 1	-1.60	0.04740
Dnajb9	predicted gene 6568; DnaJ (Hsp40) homolog, subfamily B, member 9	-1.60	0.01959
C330018D20Rik	RIKEN cDNA C330018D20 gene	-1.60	0.00958
Agl	amylo-1,6-glucosidase, 4-alpha-glucanotransferase	-1.60	0.03261
Myo10	myosin X	-1.60	0.00300
Mtmr10	myotubularin related protein 10	-1.60	0.00438
S1pr1	sphingosine-1-phosphate receptor 1	-1.60	0.02901
2610507B11Rik		-1.60	0.00201
Mcc	mutated in colorectal cancers	-1.60	0.02434
Plin4	plasma membrane associated protein, S3-12	-1.59	0.03464
Tmem182	transmembrane protein 182	-1.59	0.04115
Ap1s2	adaptor-related protein complex 1, sigma 2 subunit	-1.59	0.00231
Pdha1	pyruvate dehydrogenase E1 alpha 1	-1.58	0.03907
Pigf	phosphatidylinositol glycan anchor biosynthesis, class F	-1.58	0.03778
Tmem44	transmembrane protein 44	-1.58	0.03928
Cdh5	cadherin 5	-1.58	0.00204
Gpd2	glycerol phosphate dehydrogenase 2, mitochondrial	-1.58	0.01029
Plxna2	plexin A2	-1.58	0.03683
Trmt2b	TRM2 tRNA methyltransferase 2 homolog B (S. cerevisiae)	-1.58	0.01579
Sos2	son of sevenless homolog 2 (Drosophila)	-1.58	0.04270
Asb4	ankyrin repeat and SOCS box-containing 4	-1.57	0.04273
Prkab2	protein kinase, AMP-activated, beta 2 non-catalytic subunit	-1.57	0.02738
Hook3	hook homolog 3 (Drosophila)	-1.57	0.01375
Angpt1	angiopoietin 1	-1.57	0.02473
5830417I10Rik		-1.57	0.00040
Ddo	D-aspartate oxidase	-1.56	0.03323
Cacna1s	calcium channel, voltage-dependent, L type, alpha 1S subunit	-1.56	0.02132
Pitx2	paired-like homeodomain transcription factor 2	-1.56	0.00652
Serac1	serine active site containing 1	-1.56	0.00721
Lnx1	ligand of numb-protein X 1	-1.56	0.00302

Ankrd28	ankyrin repeat domain 28	-1.56	0.04880
Entpd5	ectonucleoside triphosphate diphosphohydrolase 5	-1.56	0.00421
Adarb1	adenosine deaminase, RNA-specific, B1	-1.56	0.03146
Cmtm4	CKLF-like MARVEL transmembrane domain containing 4	-1.56	0.01916
4930539J05Rik		-1.56	0.00572
Ulk2	Unc-51 like kinase 2 (C. elegans)	-1.56	0.04852
Col24a1	collagen, type XXIV, alpha 1	-1.55	0.04894
Gpld1	glycosylphosphatidylinositol specific phospholipase D1	-1.55	0.02023
Heatr5a	HEAT repeat containing 5A	-1.55	0.02115
Fam210a	family with sequence similarity 210 member A	-1.55	0.00161
Asb1	ankyrin repeat and SOCS box-containing 1	-1.55	0.01277
Rragd	Ras-related GTP binding D	-1.55	0.03491
Sppl2a		-1.55	0.01870
Podxl	podocalyxin-like	-1.54	0.03943
Slc16a10	solute carrier family 16 (monocarboxylic acid transporters), member 10	-1.54	0.01117
Gatsl3	GATS protein-like 3	-1.54	0.00196
B3galt1	UDP-Gal:betaGlcNAc beta 1,3-galactosyltransferase, polypeptide 1	-1.54	0.01276
Slc30a1	solute carrier family 30 (zinc transporter), member 1	-1.53	0.02545
Kalrn	kalirin, RhoGEF kinase	-1.53	0.03225
Fbxo40	F-box protein 40	-1.53	0.00201
Ggcx	gamma-glutamyl carboxylase	-1.53	0.03939
Arhgef9	CDC42 guanine nucleotide exchange factor (GEF) 9	-1.53	0.01254
Pdlim5	PDZ and LIM domain 5	-1.53	0.03316
Ppp2r3a	protein phosphatase 2 (formerly 2A), regulatory subunit B", alpha; RIKEN cDNA 3222402P14 gene	-1.53	0.00065
Tmed5	transmembrane emp24 protein transport domain containing 5	-1.53	0.00490
Mreg	melanoregulin	-1.53	0.01739
Fgf13	fibroblast growth factor 13	-1.53	0.00272
Zyg11b	zyg-ll homolog B (C. elegans)	-1.52	0.03191
Zfp759	zinc finger protein 759	-1.52	0.04706
Opa1	similar to optic atrophy 1 (autosomal dominant); optic atrophy 1 homolog (human)	-1.52	0.00205
Sypl	synaptophysin-like protein	-1.52	0.00043
Twsg1	twisted gastrulation homolog 1 (Drosophila)	-1.52	0.04662
Kcna5	potassium voltage-gated channel, shaker-related subfamily, member 5	-1.52	0.01403
Zfp106	zinc finger protein 106	-1.52	0.00047
Slc22a5	solute carrier family 22 (organic cation transporter), member 5	-1.52	0.03119
Me1	predicted gene 7049; similar to NADP-dependent malic enzyme (NADP-ME) (Malic enzyme 1)	-1.52	0.00525
Mllt3	myeloid/lymphoid or mixed-lineage leukemia (trithorax homolog, Drosophila); translocated to, 3	-1.52	0.01003
Sik1	salt inducible kinase 1	-1.51	0.00032

Disease Models & Mechanisms • Supplementary information

Phtf2	putative homeodomain transcription factor 2	-1.51	0.02256
Prickle1	prickle like 1 (Drosophila)	-1.51	0.01449
Slc9a6	solute carrier family 9 (sodium/hydrogen exchanger), member 6	-1.51	0.00218
Atad1	ATPase family, AAA domain containing 1	-1.51	0.00676
Neurl1b	predicted gene, EG240055	-1.51	0.00301
Paip2b	poly(A) binding protein interacting protein 2B	-1.51	0.00711
Neb	nebulin	-1.51	0.00929
Rtp3	receptor transporter protein 3	-1.51	0.04173
Gdpd1	glycerophosphodiester phosphodiesterase domain containing 1	-1.51	0.04404
4921531C22Rik		-1.51	0.02288
Rab10	RAB10, member RAS oncogene family	-1.50	0.00112
Fbxo3	F-box protein 3	-1.50	0.04305
Tie1	tyrosine kinase with immunoglobulin-like and EGF-like domains 1	-1.50	0.00191
Cnnm2	cyclin M2	-1.50	0.00509
Fmo5	similar to Flavin containing monooxygenase 5; flavin containing monooxygenase 5	-1.50	0.02178

Disease Models & Mechanisms • Supplementary information
